# Cancer-associated adipocytes confer CDK4/6 inhibitor resistance in ER+ breast cancer through an IL-6/STAT3/SREBF2 axis coupled with cholesterol metabolism and cell cycle reprogramming

**DOI:** 10.7150/ijbs.130653

**Published:** 2026-06-10

**Authors:** Renhong Huang, Yiwei Tong, Xi Sun, Haoyu Wang, Zheng Wang, Yujie Tang, Kunwei Shen, Xiaosong Chen

**Affiliations:** 1 Department of General Surgery, Comprehensive Breast Health Center, Ruijin Hospital, Shanghai Jiao Tong University School of Medicine, Shanghai, China.; 2 Shanghai Key Laboratory of Reproductive Medicine, Department of Histoembryology, Genetics and Developmental Biology, Shanghai Jiaotong University School of Medicine, Shanghai, China.

## Abstract

Cancer-associated adipocytes (CAAs) within the tumor microenvironment (TME) critically regulate oncogenic progression. However, the mechanistic basis underlying CAAs-mediated CDK4/6 inhibitor (CDK4/6i) resistance in estrogen receptor-positive (ER+) breast cancer remains elusive. In this study, we revealed that CAAs supernatant demonstrated enhanced capacity to induce CDK4/6i resistance in ER+ breast cancer cells compared to NAs-derived conditioned medium. Through integrated RNA sequencing and cytokine microarray screening, we identified marked upregulation of IL-6 in both CAAs and their conditioned media. Mechanistically, CAAs-derived IL-6 activates the JAK-STAT3 axis, leading to transcriptional upregulation of SREBF2, which directly drives CDK4/6i resistance through HMGCR-mediated lipid metabolism and CDKN2C-mediated cell cycle progression (IL-6**-**STAT3**-**SREBF2-HMGCR/CDKN2C axis). Reciprocally, breast cancer cell-secreted exosomal miR-1246 promotes the transformation of normal adipocytes (NAs) to CAAs via PAX5-dependent regulation, and CAAs highly express UCHL1, which stabilizes KLF5 through K48-linked deubiquitination to activate NF-κB signaling, thereby augmenting IL-6 production (exosomal miR-1246-PAX5 and UCHL1-KLF5-NF-κB loop). Pharmacologic inhibition of HMGCR with simvastatin, alone or combined with IL-6 blockade, restored CDK4/6i sensitivity in vitro and in vivo, highlighting a clinically accessible strategy to overcome adipocyte-mediated resistance. Collectively, our findings establish that CAAs confer CDK4/6i resistance in ER+ breast cancer through the IL-6-driven SREBF2 activation axis, sustained by a reciprocal exosomal miR-1246/UCHL1-mediated feedback loop.

## Introduction

Estrogen receptor-positive (ER+) breast cancer constitutes the predominant molecular subtype of breast malignancy, comprising approximately 70% of incident cases worldwide 1. The therapeutic landscape for ER+ breast cancer has been fundamentally transformed by the clinical implementation of cyclin-dependent kinase 4/6 inhibitors (CDK4/6i), now established as first-line therapeutic mainstays in combination with endocrine therapies. Nevertheless, the durable clinical efficacy of CDK4/6i regimens is progressively undermined by evolving intrinsic and acquired resistance mechanisms, highlighting the urgent imperative to delineate the molecular determinants of resistance pathogenesis 2.

The development of resistance to CDK4/6i is primarily mediated through tumor-intrinsic adaptive mechanisms, including compensatory CDK2-Cyclin E activation, RB1 functional loss, and hyperactivation of alternative signaling pathways 3-10, novel post-translational modifications, such as O-GlcNAcylation of the transcription factor MITF, along with genomic alterations in ESR1 and TP53^10^. Although CDK4/6i have achieved clinical success in ER+ breast cancer, therapeutic resistance persists as a major challenge, with tumor progression often arising through adaptive mechanisms within the tumor microenvironment (TME) 11, 12. Adipocytes, as the most abundant cellular component in adipose tissue, play essential roles in energy storage, tumor progression, immune regulation, and drug sensitivity 13-15. Through tumor-derived factors and cell-cell interactions, peritumoral normal adipocytes (NAs) are reprogrammed into CAAs, acquiring protumorigenic properties 16, 17. CAAs function as secretory hubs, releasing multiple cytokines that enhance malignant properties including tumor stemness, proliferative capacity, angiogenic potential, invasive behavior, metastatic competence, and therapeutic resistance 18, 19. Thus, a deeper understanding of TME-mediated resistance mechanisms, particularly adipocyte-tumor crosstalk, is critically needed to identify novel therapeutic targets and develop strategies for restoring CDK4/6i sensitivity in treatment-refractory breast cancers. Emerging evidence implicates dysregulated cholesterol homeostasis in resistance to targeted therapies, including CDK4/6i 20. Activation of the sterol regulatory element-binding transcription factor 2 (SREBF2) drives expression of 3-hydroxy-3-methylglutaryl-CoA reductase (HMGCR), the rate-limiting enzyme in cholesterol biosynthesis, and this pathway has been linked to therapeutic resistance in several malignancies 21, 22. Notably, pharmacological inhibition of HMGCR with statins—widely used, well-tolerated, and inexpensive agents, has been shown to restore sensitivity to various anticancer treatments in preclinical models, suggesting a readily translatable strategy 23. However, whether targeting the SREBF2-HMGCR axis can overcome acquired resistance to CDK4/6i, particularly in the context of a pro-tumorigenic microenvironment, remains unexplored.

This study investigates the contribution of CAAs to CDK4/6i resistance in ER+ breast cancer and delineates the underlying molecular mechanisms. We systematically characterize the bidirectional crosstalk and compensatory signaling between ER+ tumor cells and CAAs, with the translational objective of identifying co-targeting strategies that concurrently address both malignant cells and their adipocyte-rich microenvironment. These dual-targeting approaches may overcome acquired resistance and restore CDK4/6i sensitivity, potentially improving clinical outcomes for patients with advanced ER+ breast cancer.

## Materials and Methods

### Patients and adipocyte tissue samples

In this study, CAAs and NAs tissue samples, along with tumor and normal mammary gland specimens and serum samples, were obtained from breast cancer patients and benign tumor cases undergoing surgery at the Comprehensive Breast Health Center, Ruijin Hospital, Shanghai Jiao Tong University School of Medicine between September 2020 and September 2023. All participants provided written informed consent after receiving complete information about the study objectives. The study protocols and procedures were reviewed and approved by the Institutional Review Board of Ruijin Hospital, Shanghai Jiao Tong University School of Medicine.

### Isolation and induction of adipocytes

NAs and CAAs were isolated from tissue samples within 2 cm of tumors in patients with benign breast conditions and breast cancer, respectively, using established protocols 24. Detailed experimental methods and protocols are provided in the supplementary appendix.

### Cell culture

The human breast cancer cell lines MCF7, T47D, BT474, SKBR3, MDA-MB-231, and MDA-MB-453 were obtained from the Type Culture Collection of the Chinese Academy of Sciences (Shanghai, China) and routinely maintained in high-glucose Dulbecco's Modified Eagle Medium (DMEM) supplemented with 10% FBS and 1% penicillin/streptomycin antibiotic solution. All cell lines were cultured at 37°C in a humidified incubator with 5% CO₂ atmosphere.

### Plasmids' construction

The GFP expression vectors were sourced from the DNA library at Shanghai Jiao Tong University School of Medicine. Plasmids were constructed by Shanghai Bioegene Co.,Ltd. Following the manufacturer's protocol, we employed a lentiviral system to co-transfect HEK293T packaging cells with the GFP vector along with psPAX2 and pMD2.G packaging plasmids. The resulting GFP-overexpressing lentiviral particles were then used to generate stable GFP-expressing MCF7 and T47D cell lines through viral transduction.

### Cell viability and clone formation assays

For viability assays, cells were plated at a density of 5×10³ cells/well in 96-well plates and allowed to adhere overnight. Following experimental treatments, cell viability was assessed using the CellTiter-Glo® Luminescent Cell Viability Assay (Promega, G7573). Briefly, 50 μL of CellTiter-Glo® reagent was added to each well, after which plates were gently agitated and incubated at room temperature for 30 minutes to stabilize luminescence signals before measurement with a PerkinElmer microplate reader. For clonogenic assays, 500 cells were seeded into 6-well plates and cultured until visible colonies formed (typically >50 cells/colony). Colonies were then fixed and stained with 0.2% crystal violet for 20 minutes prior to photographic documentation and quantification.

### Bulk RNA sequencing

Bulk RNA sequencing was conducted in collaboration with Genergy Biotechnology Co., Ltd. (Shanghai, China). Differential expression analysis was performed using the DEGseq package, employing an MA-plot-based method with random sampling model to identify differentially expressed transcripts (DETs) with statistical significance (P < 0.05) and minimum fold change ≥ 2. The identified DETs were subsequently subjected to comprehensive functional annotation and pathway enrichment analysis using Gene Ontology (GO), Kyoto Encyclopedia of Genes and Genomes (KEGG), and Gene Set Enrichment Analysis (GSEA). Pathways were considered significantly enriched when meeting the criteria of P < 0.05 and containing at least two associated genes.

### Western blot

Protein extraction was performed using RIPA lysis buffer supplemented with 1% phenylmethanesulfonyl fluoride (PMSF). Protein concentrations were determined by bicinchoninic acid (BCA) assay. Equal amounts of protein lysates were resolved by sodium dodecyl sulfate-polyacrylamide gel electrophoresis (SDS-PAGE) and subsequently transferred to polyvinylidene difluoride (PVDF) membranes. After blocking with 5% bovine serum albumin (BSA) for 1 hour at room temperature, membranes were incubated overnight at 4°C with primary antibodies, followed by 1-hour incubation with horseradish peroxidase (HRP)-conjugated goat anti-rabbit secondary antibody at room temperature. Antibody details are provided in Table S2. Protein bands were visualized using a Luminescent Image Analyzer detection system (Fujifilm LAS-4000).

### RT-qPCR

Total RNA was extracted using TRIzol reagent (Invitrogen, Carlsbad, CA, USA) following the manufacturer's protocol. cDNA synthesis was performed using the PrimeScript™ RT reagent kit (Takara, formerly Thermo Fisher Scientific) with random hexamer primers. Gene expression analysis was conducted by real-time quantitative PCR (RT-qPCR) in technical triplicates using SYBR Green Master Mix (Roche) on a QuantStudio Real-Time PCR System (Applied Biosystems). All primer sequences used in this study are provided in Tables S3 and S4. Relative gene expression levels were calculated using the comparative threshold cycle (2^-ΔΔCt)^ method with normalization to housekeeping genes.

### Cleavage Under Targets and Tagmentation (CUT&Tag)

To profile genome-wide SREBF2 DNA-binding activity in MCF7 cells, we performed CUT&Tag assays using the Hyperactive Universal CUT&Tag Assay Kit for Illumina (Vazyme, TD903-02). Briefly, MCF7 cells were transfected with FLAG-tagged SREBF2 plasmid, and 24 hours post-transfection, approximately 100,000 cells were harvested for the assay. Cells were washed twice with PBS and immobilized on concanavalin A-coated beads for 10 minutes at room temperature. The beads were then incubated overnight at 4°C with either anti-FLAG antibody (Cell Researcher Biotech, A105) or rabbit IgG isotype control (Cell Signaling Technology, 2729), followed by 1-hour incubation with goat anti-rabbit IgG secondary antibody (Abcam, ab6702) at room temperature. After washing, beads were incubated with pA/G-Tn5 transposase for targeted fragmentation. Library preparation was performed following standard protocols, including DNA fragmentation and amplification steps as previously described. Final libraries were purified using VAHTS DNA Clean Beads (Vazyme, N411), quantified, and subjected to next-generation sequencing on the Illumina NovaSeq platform (Novogene).

### CHIP-seq and CHIP-qPCR

Chromatin immunoprecipitation quantitative PCR (CHIP-qPCR) was performed following established methods 18. Briefly, cells were cross-linked with 1% formaldehyde for 8 minutes at 25°C, followed by quenching with 0.125 M glycine. Chromatin was then digested using micrococcal nuclease (MNase; NEB, M0247S) and sheared by sonication (5 cycles of 20 seconds on/30 seconds off per cycle). The fragmented chromatin was immunoprecipitated by incubation with SREBF2-specific antibodies with rotation at 4°C for 12 hours, followed by capture using Protein A/G magnetic beads (Thermo Fisher Scientific) for 4 hours at 4°C. Precipitated DNA was purified and analyzed by both CHIP-seq and qPCR, with input chromatin serving as the normalization control.

### Co-immunoprecipitation (Co-IP)

To examine the potential interaction between UCHL1 and KLF5, Co-IP assays were performed. Cells were lysed in ice-cold IP buffer supplemented with protease inhibitors. Following centrifugation, 500 μg of protein lysate was pre-cleared and then incubated overnight at 4°C with 2 μg of specific antibody. Protein A/G agarose beads were added to capture immune complexes, which were then washed extensively. Bound proteins were eluted by boiling in Laemmli buffer. The eluates, along with input controls, were resolved by SDS-PAGE and transferred to PVDF membranes. Western blotting was subsequently carried out using anti-UCHL1 and anti-KLF5 primary antibodies, followed by HRP-conjugated secondary antibodies (Table S2). Signals were detected via enhanced chemiluminescence.

### Immunofluorescence (IF)

Cells were plated on glass coverslips at a density of 1×10⁶ cells per well and allowed to adhere. Antigen retrieval was performed by heat-induced epitope retrieval in citrate buffer at 121°C for 15 minutes using an autoclave. Following three PBS washes, samples were fixed with 4% paraformaldehyde for 20 minutes at room temperature and permeabilized with 0.2% Triton X-100 for 20 minutes. Non-specific binding sites were blocked with 5% bovine serum albumin (BSA) for 1 hour before incubation with primary antibodies overnight at 4°C. After washing, coverslips were incubated for 1 hour at room temperature with either goat anti-rabbit IgG or isotype control secondary antibodies (diluted 1:500 in PBS) protected from light. Nuclei were counterstained with 4',6-diamidino-2-phenylindole (DAPI) for 5 minutes, and fluorescent images were acquired using a confocal microscope equipped with appropriate filter sets.

### Hematoxylin and eosin (HE) staining

Mouse lung tissues were processed for histological analysis using a standard HE staining kit (Beyotime Biotechnology, Shanghai, China). Following 24-hour fixation in 4% paraformaldehyde at 4°C, tissues were dehydrated through a graded ethanol series (70%, 80%, 90%, and 100%), cleared in xylene, and embedded in paraffin. Serial 5-μm sections were cut using a microtome, dewaxed in xylene, rehydrated through descending alcohol concentrations, and stained with hematoxylin for 5 minutes followed by eosin for 3 minutes. Tissue morphology and metastatic nodules were examined and imaged using a light microscope equipped with a digital camera system.

### Immunohistochemistry (IHC)

Sectioned tumor tissues of the homograft model were embedded in paraffin, rehydrated, and blocked by incubation with goat serum. After rehydration, sections were blocked with goat serum before overnight incubation with primary antibodies at 4°C. HRP-conjugated goat anti-rabbit or anti-mouse secondary antibodies were then applied for 1 hour at room temperature. Visualization was achieved using Metal Enhanced DAB Substrate (Dako, Denmark) with hematoxylin counterstaining (Beyotime, China). Target protein density was quantified by measuring integrated optical density (IOD) using ImageJ software.

### Exosome labeling and co-incubation with cultured adipocyte cells

The internalization of ER+ breast cancer-derived exosomes by cultured NAs and CAAs was assessed through confocal microscopy following co-culture. Purified exosomes were fluorescently labeled with PKH67 (Sigma, Germany) according to established protocols prior to the experiment 25.

### Establishment of mice model

Comprehensive experimental methods and protocols can be found in the supplementary appendix.

### Cytokine microarray

Protein profiling was conducted using the Human Obesity Antibody Array (Raybiotech, Norcross, GA, USA) for semi-quantitative analysis of 62 human proteins. Following manufacturer's protocol, arrays were initially blocked with 2 ml blocking buffer per well for 30 minutes at room temperature. Subsequently, 1 ml cell supernatant was applied to each well and incubated overnight at 4°C. Arrays underwent sequential washing with Wash Buffer I and Wash Buffer II, followed by 2-hour incubation with 1 ml biotinylated antibody cocktail at 25°C. Detection was achieved through 2-hour room temperature incubation with HRP-streptavidin, with signal visualization performed using chemiluminescence imaging membranes (Pierce Biotechnology). Differentially secreted cytokines were defined as those with signal intensity difference >1.5-fold between CAA and NA supernatants.

### Enzyme-linked immunosorbent assay (ELISA)

The concentrations of secreted proteins, including IL-6, CCL2, and CXCL5 (R&D Systems, Minneapolis, MN, USA) in NAs and CAAs supernatants were quantified using manufacturer-recommended sandwich ELISA protocols. Protein detection signals were acquired using a GenePix microarray laser scanning system (Molecular Devices, USA).

### Establishment of patient-derived organoids (PDOs)

Human ER+ organoids were cryopreserved in our biobank following an established protocol 26. All reagents for patient-derived organoid (PDO) culture and experimental procedures were implemented as described in previous publications 27.

PDOs were defined as CDK4/6i-sensitive if organoid diameter was reduced by ≥50% and viable organoid number decreased by ≥60% after 5 days of ribociclib treatment; PDOs were defined as CDK4/6i-resistant if organoid diameter and viable count were reduced by <20% under identical treatment.

### Statistical analysis

All experiments were conducted with a minimum of three biological replicates, with results presented as mean ± standard deviation (SD). Intergroup comparisons were analyzed using Student's t-test, one-way ANOVA, non-parametric test or Mann-Whitney U, as appropriate. Statistical computations were performed using GraphPad Prism (version 6.02; GraphPad Software, San Diego, CA) and SPSS Statistics (version 21.0; IBM, Armonk, NY). Statistical significance was defined as P < 0.05.

## Results

### CAA-secreted IL-6 drives CDK4/6i resistance

**Figure 1:**
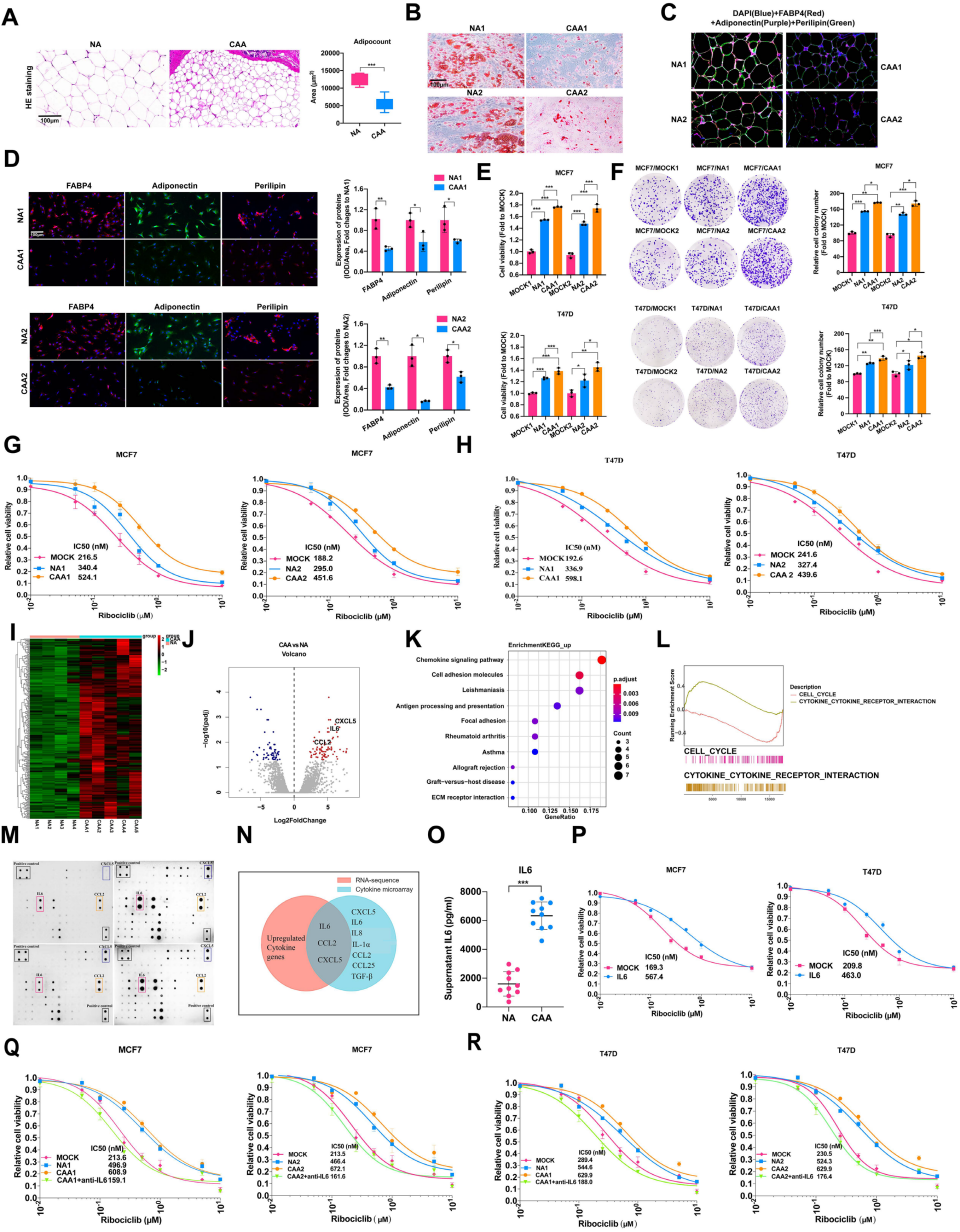
** CAAs-derived IL-6 confers CDK4/6i resistance of ER-positive breast cancer.** (A) The Hematein Eosin (HE) staining of adipocytes in NAs and CAAs. The adipocyte areas were calculated by the adipocyte counting system (AdipoCount). (B) Oil staining of NAs and CAAs after induction with induced differentiation for 28 days (Induced solution A: 1mlF12+6μl insulin+1μl IBMX+0.1μl Dex; Induced B solution: 1ml F12+6μl insulin). (C) Multiplex IHC detects the expression of adipogenic differentiation markers in NA and CAA tissues. (D) IF and statistical analysis of FABP4, Perilipin, Adiponectin in adipocytes of NAs and CAAs. (E) The cell viability and statistical analysis of MCF7 and T47D cells after treated with NAs or CAAs supernatant for 7 days. (F) Cell colony assay analysis and statistical analysis depicts the impact of NAs or CAAs supernatant on cell formation after treated with NAs or CAAs supernatant for 14 days. (G-H) CellTiter-Glo cell viability assay showed the survival rate and statistical analysis of Half-Maximal Inhibitory Concentration (IC50) of MCF7 and T47D cells induced with various concentrations of Ribociclib after treated with NAs or CAAs supernatant for 7 days. (I) Heatmap depicting the differential transcriptomic expression with RNA sequence data in NAs and CAAs primary cells. (J) Volcano plots showing DEGs of NAs and CAAs. (K) KEGG enrichment scatter plot for upregulated gene in CAAs, compared with NAs. (L) GSEA analysis for upregulated gene in CAAs, compared with NAs. (M) 62 kinds of cytokines in the supernatant of NAs and CAAs were determined semi-quantitatively by the protein microarray technology. (N) The intersection of transcriptional sequencing analysis and protein microarray data reveals the co-up-regulated cytokines of IL-6, CCL2 and CXCL5. (O) ELISA detected IL-6 secretory proteins in 10 NAs and 10 CAAs supernatants. (P-R) Cell viability assay depicted the survival rate and statistical analysis of IC50 of MCF7 and T47D cells induced with various concentrations of Ribociclib after treated with NAs or CAAs supernatants depicted IL-6 by specific antibody (10 ug/ml) for 7 days. The experiments were repeated twice with three biological replicates per experiment. All results are expressed as mean ± SD. * *P*<0.05, ** *P*<0.01, *** *P*<0.001. Mann-Whitney U test (Fig. 1A); independent two-sample Student's t test (Fig. 1D, 1O); One-way ANOVA followed by Tukey's test (Fig. 1E-F).

We observed an inverse correlation between CDK4/6i resistance and adipogenic differentiation capacity. Quantitative analysis of NAs and CAAs from CDK4/6i-resistant patients revealed that peritumoral adipocytes displayed significantly reduced cross-sectional area and irregular morphology (Figure 1A). CAAs accumulated substantially fewer lipid droplets following 28-day differentiation induction (Oil Red O staining), and expressed lower levels of adipogenic markers (FABP4, adiponectin, perilipin) compared to NAs (Figure 1B-D, Supplementary Figures S1-S2). These findings collectively demonstrate that CAAs acquire a dedifferentiated phenotype characterized by compromised lipid storage capacity and reduced expression of adipocyte-specific markers.

To further investigate the consequences, we treated ER+ breast cancer cells (MCF7, T47D) with conditioned media from NAs or CAAs. Both enhanced proliferation of MCF7 and T47D cell lines (Figure 1E), with similar effects observed in MDA-MB-231 cells (Supplementary Figure S3A). In contrast, these adipocyte-conditioned media showed no proliferative effect on MDA-MB-453, SKBR3, or BT474 cell lines (Supplementary Figure S3A). Colony formation assays corroborated these findings (Figure 1F, Supplementary Figure S3B). Both NA- and CAA-derived factors induced resistance to ribociclib (with CAAs showing stronger effects, Figure 1G-H) and also conferred resistance to palbociclib, fulvestrant, and 4-hydroxytamoxifen in ER+ cells (Supplementary Figure S6). Fractionation of conditioned media revealed that soluble cytokines, rather than exosomes, mediated these proliferative and resistance-conferring effects (Supplementary Figures S4, S5).

We conducted RNA sequencing of NAs and CAAs (Figure 1I-J). KEGG pathway analysis of differentially expressed genes (DEGs) showed significant enrichment in chemokine signaling pathways and cell adhesion molecules (Figure 1K). Gene set enrichment analysis (GSEA) further demonstrated activation of the cytokine-cytokine receptor interaction pathway in CAAs compared to NAs (Figure 1L).

To screen CAAs-secreted cytokines that mediate CDK4/6i resistance in ER+ breast cancer cells, we performed cytokine array analysis profiling 62 soluble factors (Supplementary Table S1). Multiple cytokines including CXCL5, IL-1α, IL-6, IL-8, CCL2, CCL25 and TGF-β were markedly upregulated in CAA-conditioned medium relative to NA-conditioned medium (Figure 1M). Integration with transcriptomic data identified IL-6, CCL2, and CXCL5 as the most differentially expressed cytokines (Figure 1N), which was further validated by quantitative secretion assays (Figure 1O, Supplementary S7A). Functional assays confirmed recombinant IL-6 markedly attenuated CDK4/6i cytotoxicity, while CCL2 and CXCL5 showed no such effect (Figure 1P, Supplementary Figure S7). Further functional characterization demonstrated that only recombinant IL-6 significantly attenuated CDK4/6i cytotoxicity (Figure 1Q-R). These findings establish IL-6 as the predominant CAAs-derived cytokine driving CDK4/6i resistance in ER+ breast cancer.

### CAAs mediates CDK4/6i resistance through SREBF2-dependent cell cycle regulation and lipid metabolic reprogramming

**Figure 2:**
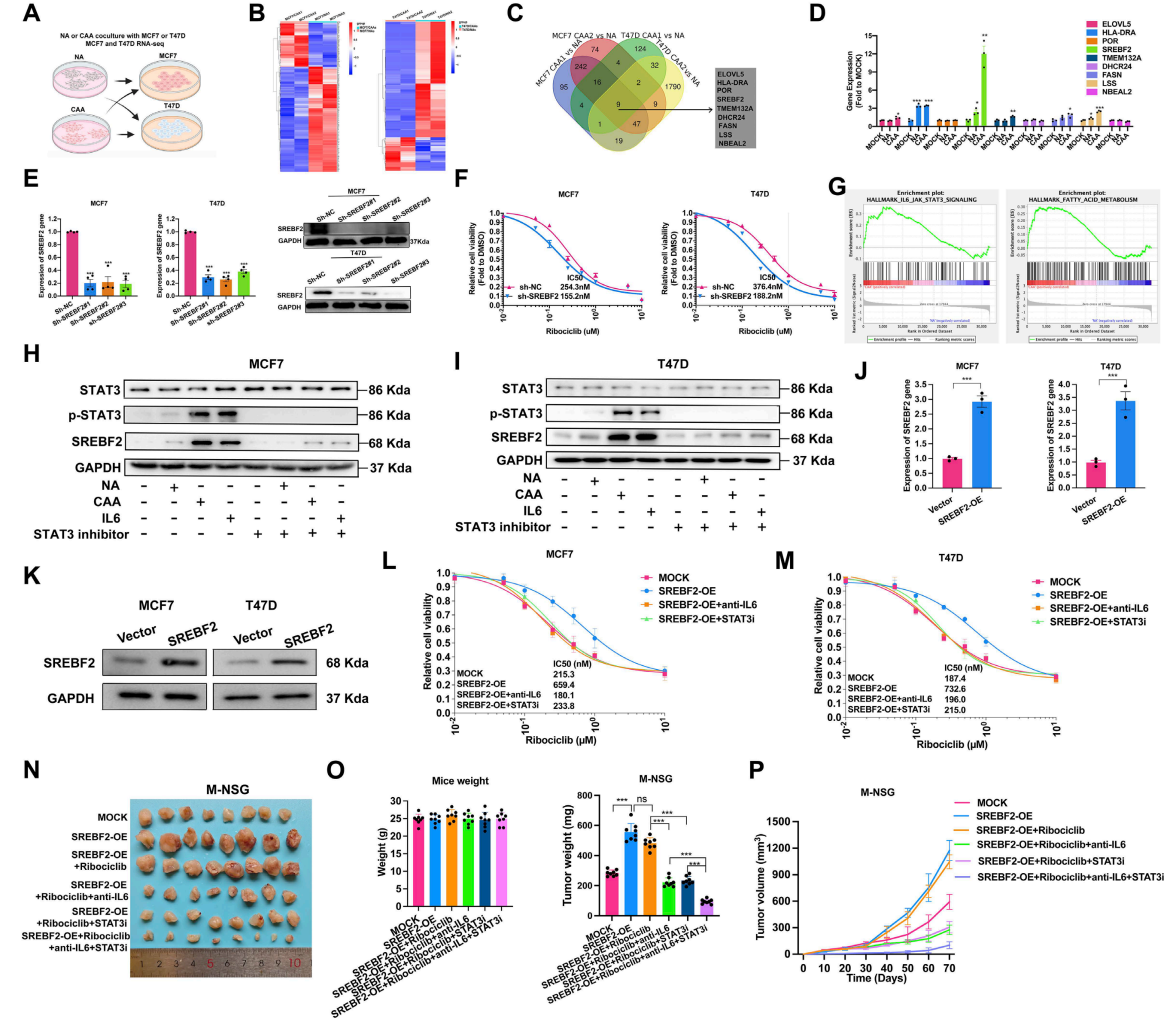
** CAAs-derived IL-6 upregulated the expression of SREBF2, which leads to CDK4/6i resistance.** (A-B) Cells were treated with NA or CAAs supernatants for 7 days, and the transcriptome sequencing analysis were performed, and the heatmap depicted the DEGs. (C) Venn plot showed up-regulated genes after breast cancer cells treated with CAAs supernatants, compared with NA supernatants. (D) The expression of up-regulated genes in MCF7 cell lines was determined by qPCR after treatment with NAs or CAAs supernatant. (E) qPCR and Western blotting were applied to detect SREBF2 expression after breast cancer cell lines were transfected with vector control and the SREBF2-knockdown plasmid. (F) Cell viability and the IC50 of cells with or without SREBF2-knockdown induced with various concentrations of Ribociclib. (G) GSEA enrichment analysis of signaling pathway in breast cancer cells were activated after treated with CAAs supernatants, compared with NA supernatants. (H-I) Proteins STAT3, p-STAT3 and SREBF2 were measured with or without the treatment of NAs supernatants, CAAs supernatants, IL-6 and STAT3 inhibitor in MCF7 and T47D cells. (J-K) To overexpress SREBF2, the SREBF2 gene was synthesized and cloned into the pcDNA3.1(+) vector, the expression of SREBF2 gene and protein were assessed by RT-qPCR and Western blot. (L-M) Cell viability and the IC50 in MCF7 and T47D cells under conditions of SREBF2-overexpression, followed by treatment with IL-6 or the STAT3 inhibitor. (N) Images of subcutaneous mammary fat pad tumors in M-NSG mice in different groups. Tumors were harvested after 1.0×10^7^ MCF7 cells with or without SREBF2 overexpression per mouse were injected into the subcutaneous mammary fat pad of the M-NSG mice. The mice were injected with either the anti-IL-6 antibody (10 μg once) and/or STAT3 inhibitor TTI-101(100 mg/kg once) by oral gavage, which scheduled every two days. (O-P) Mice weights and tumor weights of the M-NSG mice were recorded at the time of sacrifice, and a continuous line graph were applied to compare tumor growth in each group. The experiments were repeated twice with three biological replicates per experiment. All results are expressed as mean ± SD. * *P*<0.05, ** *P*<0.01, *** *P*<0.001. One-way ANOVA followed by Tukey's test (Fig. 2D-E, 2O); independent two-sample Student's t test (Fig. 2J).

To elucidate the mechanisms underlying adipocyte-mediated CDK4/6i resistance, we treated ER+ breast cancer cells with NA and CAA conditioned medium and conducted RNA-seq (Figure 2A-B). Transcriptomic screening identified nine significantly upregulated lipid metabolism-related genes, among which SREBF2 was markedly elevated in CAA medium-exposed cells (Figure 2C-D).

SREBF2 depletion significantly enhanced CDK4/6i sensitivity (Figure 2E-F), while Gene Set Enrichment Analysis (GSEA) revealed concomitant activation of both IL-6/JAK/STAT3 signaling and fatty acid metabolism pathways (Figure 2G). Both NA/CAA conditioned medium and recombinant IL-6 upregulated SREBF2, and STAT3 inhibition blocked this process, proving CAA-derived IL-6 controls SREBF2 through the IL-6-JAK-STAT3 axis (Figure 2H-I, Supplementary Figure S8). We constructed SREBF2-overexpressing ER+ breast cancer cells (Figure 2J-K). SREBF2 overexpression induced CDK4/6i resistance, which was rescued by IL-6 or STAT3 pathway suppression (Figure 2L-M).* In vivo* experiments further confirmed that SREBF2-mediated CDK4/6i resistance could be eliminated by anti-IL-6 drugs or STAT3 inhibitors (Figure 2N-P).

**Figure 3:**
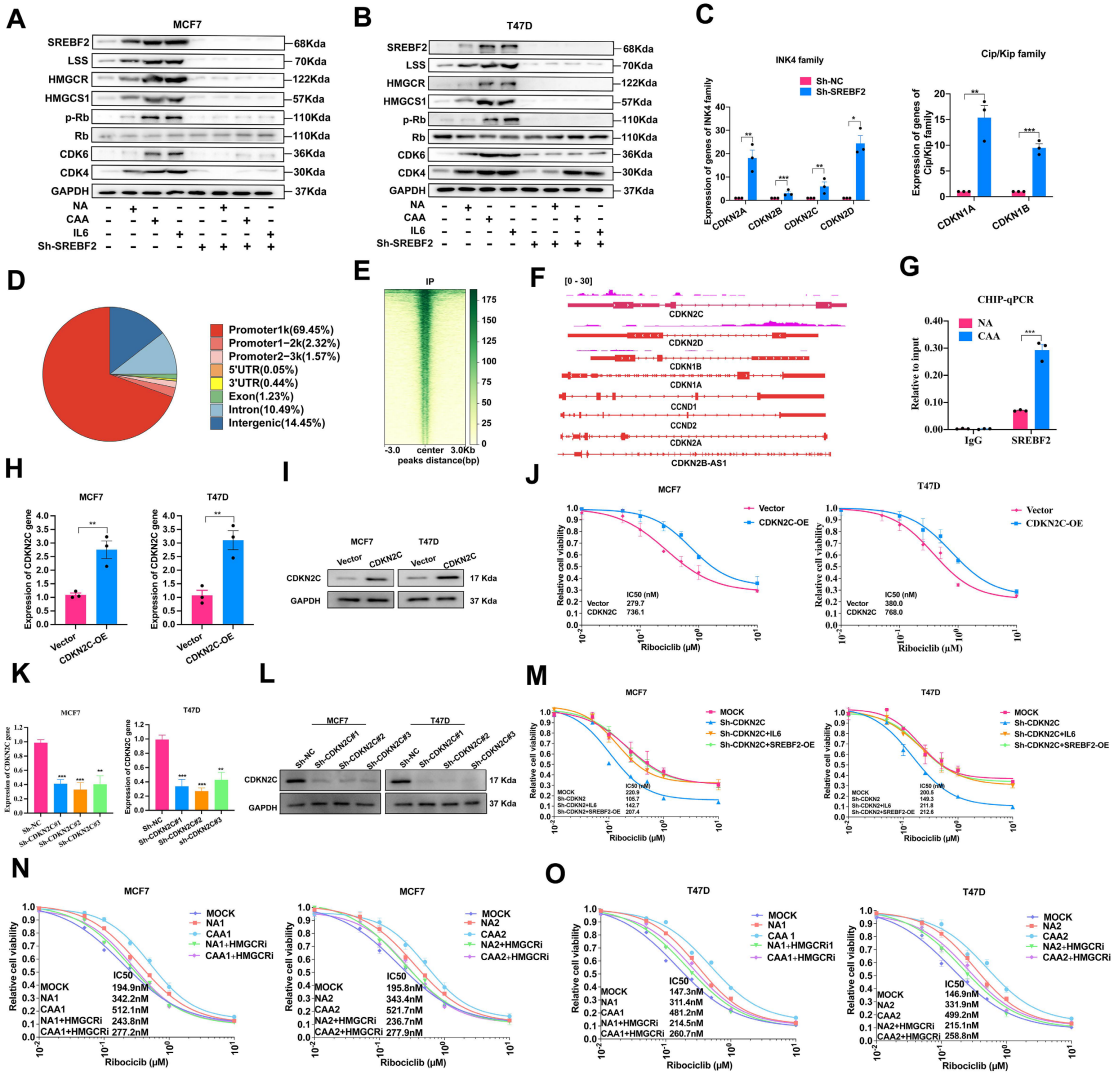
** CAAs-derived IL-6 promote CDK4/6i resistance by mediating cell cycle and lipid metabolism.** (A-B) Proteins SREBF2, LSS, HMGCR, HMGCS1, and CDK4, CDK4, Rb and p-Rb were detected with or without the treatment of NA supernatants, CAAs supernatants, IL-6 and SREBF2-knockdown. (C) Expression of INK4 family (CDKN2A (p16), CDKN2B (p15), CDKN2C (p18), CDKN2D (p19)) and Clip/Kip family (CDKN1A (p21), CDKN1B (p27)) were evaluated in MCF7 parental cells and SREBF2-knockdown cells. (D) The percentage of the SREBF2 peaks found within each region of the genome is shown. (E) Heatmaps showing SREBF2 CUT&Tag signals in MCF7. (F) UCSC genome tracks of SREBF2 enrichment at CDKN2C promoter loci in MCF7 based on normalized CUT&Tag read coverage. (G) CHIP-qPCR was used to verify SREBF2 binding to CDKN2C (p18). (H-I) To overexpress CDKN2C, the CDKN2C gene was synthesized and cloned into the pcDNA3.1(+) vector, the expression of CDKN2C gene and protein were assessed by RT-qPCR and Western blot. (J) Cell viability and the IC50 of cells with or without CDKN2C-overexpression induced with various concentrations of Ribociclib. (K-L) qPCR and Western blotting were applied to detect CDKN2C expression after breast cancer cell lines were transfected with vector control and the CDKN2C-knockdown plasmid. (M) Cell viability and the IC50 in cells under conditions of CDKN2C-knockdown or SREBF2 overexpression, followed by treatment with or without IL-6. (N-O) CellTiter-Glo cell viability assay showed the survival rate and statistical analysis of IC50 of MCF7 and T47D cells induced with various concentrations of Ribociclib after treated with NAs or CAAs supernatant with or without the application of HMGCR inhibitor Simvastatin (10 nM) for 7 days. The experiments were repeated twice with three biological replicates per experiment. All results are expressed as mean ± SD. * *P*<0.05, ** *P*<0.01, *** *P*<0.001. One-way ANOVA followed by Tukey's test (Fig. 3K); independent two-sample Student's t test (Fig. 3C, 3G, 3H).

Cell cycle analysis confirmed NAs/CAAs-conditioned media and IL-6 could promote cell cycle progression, while these effects could be abolished by SREBF2 knockdown (Figure 3A-B, Supplementary Figure S9). To explore its regulatory mechanism, we detected Ink4 and Cip/Kip family genes, and found SREBF2 depletion significantly upregulated cyclin D2 and these family members (Figure 3C). We performed CUT&Tag sequencing, yielding approximately 49.7 million paired end reads (~6.92 Gb). Analysis revealed 3,833 significant SREBF2-binding peaks distributed across genomic regions, with their heatmap and genomic distribution illustrated in Figure 3D-E. These peaks were enriched in promoters of cell cycle-related genes, and CDKN2C was screened as a core candidate. CUT&Tag-seq and ChIP-qPCR verified the direct binding of SREBF2 to CDKN2C promoter, confirming CDKN2C as a functional target regulating cell cycle and drug resistance (Figure 3F-G), indicating CDKN2C could be served as a novel SREBF2 target gene to mediating the drug resistance and cell cycle progression. Next, we generated ER-positive cell lines with CDKN2C overexpression (Figure 3H-I) and with CDKN2C knockdown (Figure 3K-L). Overexpression of CDKN2C conferred resistance to CDK4/6i (Figure 3J). Furthermore, the CDK4/6i-resistant phenotype induced by IL-6 or SREBF2 overexpression was reversed upon CDKN2C knockdown (Figure 3M). Given the established role of HMGCR as a canonical target of SREBF2 in cholesterol biosynthesis 28, its inhibitor simvastatin restored CDK4/6i sensitivity in cells cultured with NA/CAA medium (Figure 3N-O), suggesting that targeting the SREBF2-HMGCR axis may represent a viable strategy to overcome adipocyte-mediated drug resistance. Collectively, CAA-secreted IL-6 promotes CDK4/6i resistance through SREBF2-dependent cell cycle regulation and lipid metabolic reprogramming.

**Figure 4:**
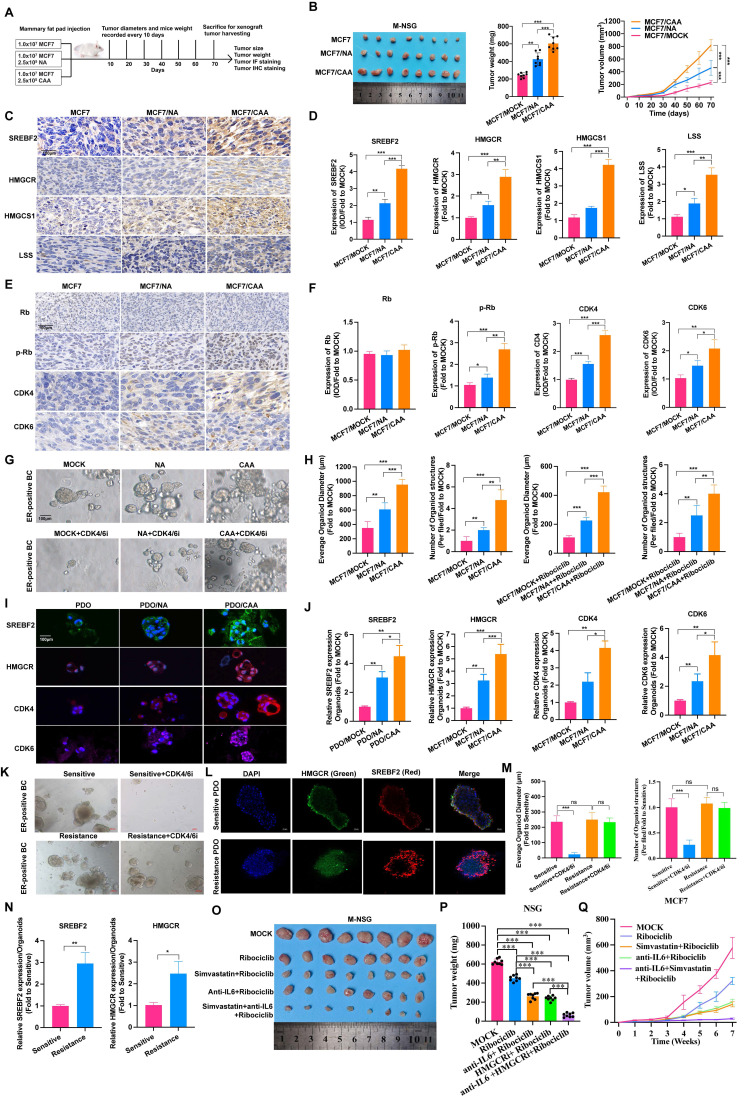
** CAAs promote cell cycle and lipid metabolism *in vivo*.** (A) Schematic diagrams of M-NSG mice model. (B) Images of subcutaneous mammary fat pad tumors in M-NSG mice in the MCF7 (n=8), MCF7/NA (n=8) or MCF7/CAA (n=8) groups. (C-D) IHC staining of proteins, SREBF2, HMGCR, HMGCS1 and LSS in each group. (E-F) IHC staining of proteins, Rb, p-Rb, CDK4, and CDK6 in each group. (G-H) Lump of a breast cancer patient with ER+/PR+/HER2- was used to form 3D-organoids in culture. The organoids were co-cultured with NAs and CAAs for 7 days. The organoids were co-cultured with NAs and CAAs for 7 days and then treated with IC50 concentration of Ribociclib for 5 days. (I-J) IF staining of SREBF2, HMGCR, CDK4 and CDK6 in the PDO, PDO/NA and PDO/CAA groups. (K, M) Lump of breast cancer patients with CDK4/6i sensitive and resistance were obtained to form 3D-organoids. Images are shown of organoids after treated with or without 200nM Ribociclib. (L, N) IF staining of SREBF2 and HMGCR in CDK4/6i sensitive or resistance PDOs. (O) Images of subcutaneous mammary fat pad tumors in M-NSG mice in different groups. The mice were injected with either the anti-IL-6 antibody (10 μg once) and/or Simvastatin (10 mg/kg once) by oral gavage, which scheduled every two days. (P-Q) Tumor weights of the M-NSG mice were recorded at the time of sacrifice, and a continuous line graph were applied to compare tumor growth in each group of the M-NSG mice. The experiments were repeated twice with three biological replicates per experiment. * *P*<0.05, ** *P*<0.01, *** *P*<0.001. One-way ANOVA followed by Tukey's test (Fig. 4B, 4D, 4F, 4H, 4J, 4M, 4P); independent two-sample Student's t test (Fig. 4N).

In MCF7 subcutaneous xenograft model, co-injection of CAAs markedly facilitated tumor growth relative to NAs (Figure 4A-B). Mechanistically, CAAs activate lipid metabolism and promote cell cycle progression, as evidenced by upregulated expression of SREBF2, HMGCR, HMGCS1 and LSS (Figure 4C-D, Supplementary Figure S10A), along with cell cycle proteins, CDK4, CDK6, Rb, and p-Rb levels (Figure 4E-F, Supplementary Figure S10B). Patient-derived organoid (PDO) models of ER-positive breast cancer were applied for clinical verification. Consistent with in vivo outcomes, CAA co-culture elevated the expression of above key proteins, enhanced PDO proliferation and induced ribociclib resistance (Figure 4G-J), validating CAA-mediated cell cycle disorder and lipid metabolism activation. PDOs were established from CDK4/6i-sensitive and resistant breast cancer specimens. Drug treatment induced distinct phenotypic responses between two groups, and immunofluorescence staining revealed higher SREBF2 and HMGCR levels in resistant PDOs (Figure 4K-N). In addition, we established M-NSG mouse model bearing MCF7 cells treated with either Simvastatin or/and anti-IL-6 antibody. Consequently, monotherapy with either Simvastatin or anti-IL-6 antibody increased CDK4/6i drug sensitivity and combined treatment achieved superior efficacy (Figure 4O-Q). Together, these data support a model in which CAA-secreted IL-6 promotes resistance to CDK4/6 inhibition through STAT3-mediated activation of SREBF2, thereby driving lipid metabolic reprogramming and cell cycle progression to sustain tumor growth.

### Exosomal miR-1246 mediates NAs cellular reprogramming by targeting PAX5, resulting in elevated IL-6 expression

**Figure 5:**
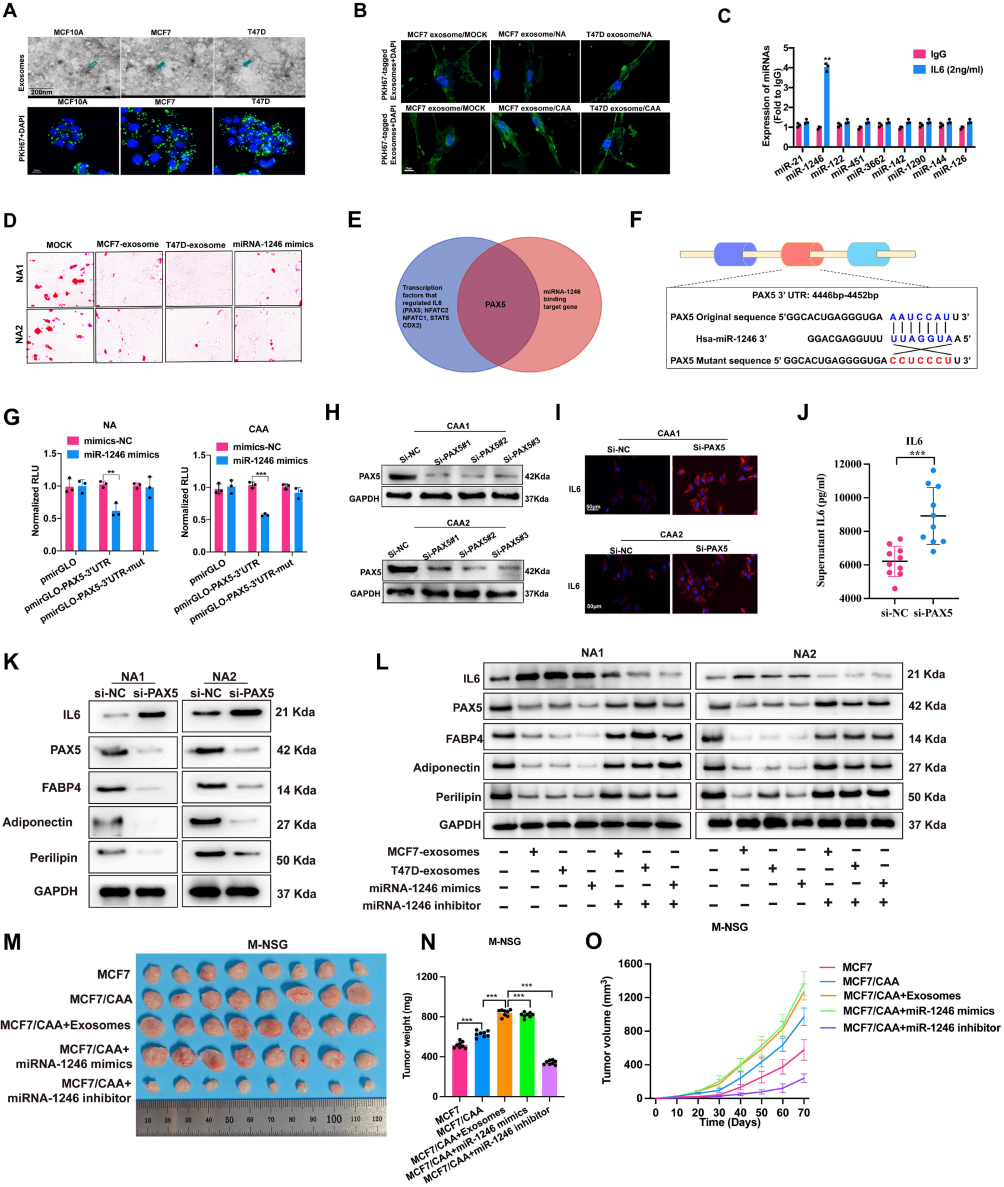
** Tumor cell-derived exosomal miRNA-1246 reprograms NAs into CAAs.** (A) Exosomes isolation from cell culture supernatants were visualized by transmission electron microscopy, and PKH67 staining was used to label exosomes. (B) Up take of PKH67 labeled exosomes by NAs and CAAs. (C) The expression of miRNAs in NA cell after treated with IgG and IL-6 (2ng/ml). (D) Red oil staining of NA1 and NA2 cells with the treatment MCF7-exosome, T47D exosome and miR-1246 mimics accompanied by the induction with induced differentiation for 28 days. (E) The intersection of transcriptional factors regulating IL-6 and miR-1246 binding targeting genes reveals PAX5 might be the gene that targeting IL-6. (F) Schematic diagram of the dual luciferase miR-1246 target reporter vector, the wild type and mutant PAX5 3′-UTR sequences are shown with the miR-1246 sequence. (G) Luciferase reporter assays of NAs and CAAs transfected with miR-1246 mimics and the wild type or mutant PAX5 reporter. (H) Western blotting analysis was used to detect PAX5 expression after breast cancer cell lines were transfected with negative control siRNA (si-NC) or siRNA targeting to PAX5 (si-PAX5). (I) IF staining of PAX5 in si-NC and si-PAX5 groups. (J) The levels of IL-6 were detected in the supernatants of 10 NAs and CAAs with or without PAX5 knockdown. (K) Western blot of the expression of FABP4, perilipin, adiponectin, IL-6 and PAX5 in si-NC and si-PAX5 groups. (L) Western blot of the expression of FABP4, perilipin, adiponectin, IL-6 and PAX5 in NA cells with the treatment of MCF7 exosomes, T47D exosomes, miR-1246 mimics, with or without miR-1246 inhibitor. (M) Images of subcutaneous mammary fat pad tumors in M-NSG mice in the MCF7, MCF7/CAA, MCF7/CAA with the treatment of exosomes isolation from MCF7 cell culture supernatants, MCF7/CAA with the treatment with miRNA-1246 mimics or inhibitor. (N-O) Tumor weights of the M-NSG mice were recorded at the time of sacrifice, and a continuous line graph were applied. The experiments were repeated twice with three biological replicates per experiment. ** *P*<0.01, *** *P*<0.001. independent two-sample Student's t test (Fig. 5C, 5G, 5J); One-way ANOVA followed by Tukey's test (Fig. 5N).

Accumulating evidence reveals aberrant tumor-derived miRNAs modulate ECM remodeling, stem cell characteristics and cell reprogramming. We first identified exosome morphology via cryo-TEM, and PKH67-stained exosomes were successfully internalized by NAs in co-culture (Figure 5A-B). Building on previous RNA-seq data, miR-1246 was significant markedly upregulated following IL-6 treatment (Figure 5C). To assess miR-1246's role in adipocyte reprogramming, we treated NAs with ER+ BC-derived exosomes or miR-1246 mimics, both of which reduced lipid droplet accumulation (Figure 5D). Bioinformatic prediction and experimental verification confirmed PAX5 as a direct target of miR-1246 that governs IL-6 release (Figure 5E-G). PAX5 silencing distinctly elevated IL-6 secretion (Figure 5H-K, Supplementary Figure S11). Exosomes and miR-1246 mimics suppressed PAX5 and adipocyte markers while boosting IL-6 level, and miR-1246 inhibitor could reverse these alterations (Figure 5L, Supplementary Figures S12-S13). Moreover, in M-NSG mice bearing MCF7 cells and CAAs, additional treatment with exosomes isolated from MCF7 culture supernatants, or with miR-1246 mimics, promoted tumor growth, whereas miR-1246 inhibitor treatment suppressed it (Figure 5M-O). In summary, ER-positive breast cancer cell-secreted exosomes deliver miR-1246 to reprogram adipocytes. miR-1246 inhibits PAX5 expression, thereby restraining adipocyte differentiation and facilitating IL-6 secretion.

### UCHL1 regulates IL-6 expression by stabilizing KLF5 through K48-linked deubiquitination

**Figure 6:**
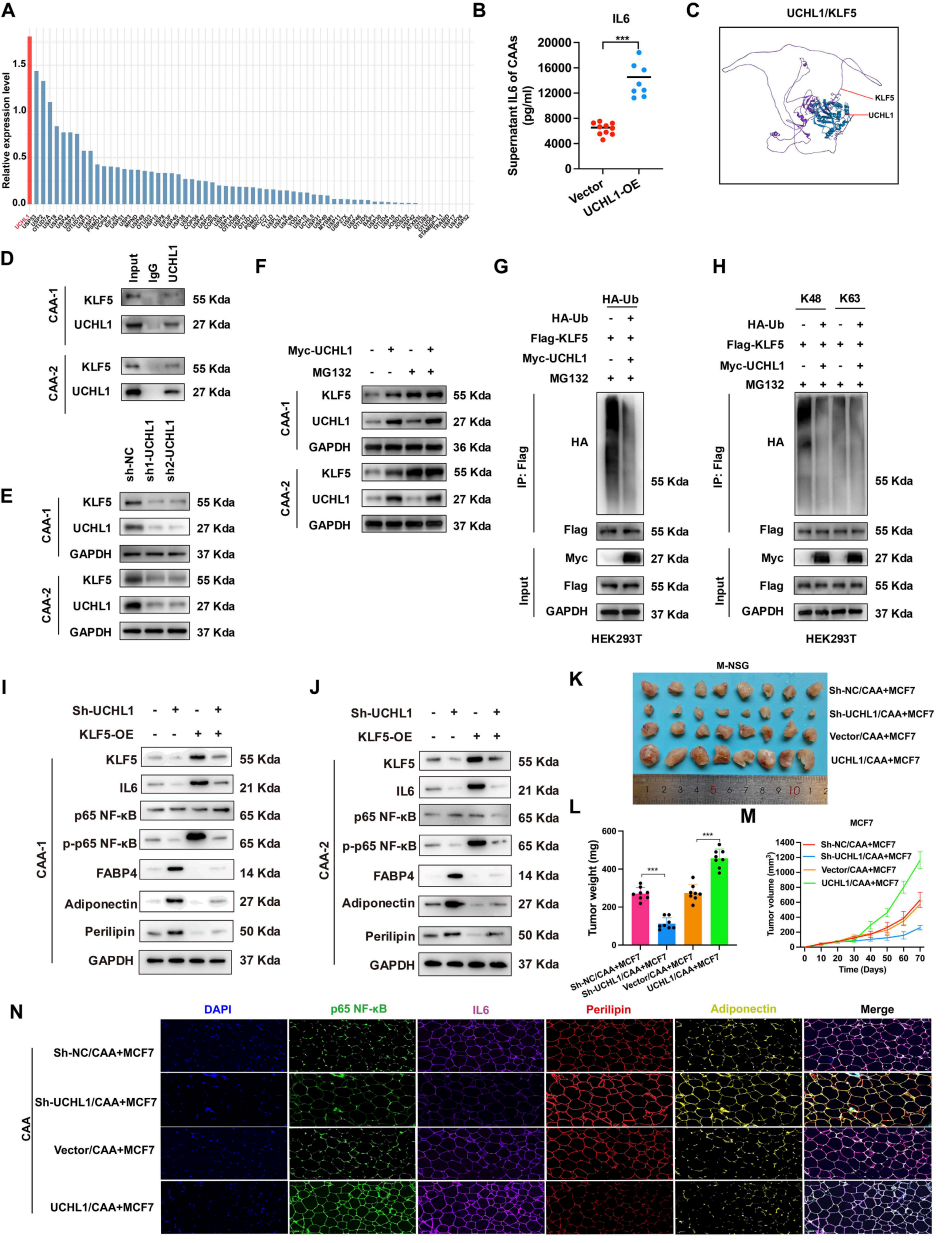
** Higher expression of UCHL1 in CAA increases IL-6 secretion by stabilizing KLF5 through K48-linked deubiquitination.** (A) Quantitative analysis of deubiquitinases (DUBs) gene expression levels in CAAs, compared with NAs. (B) ELISA detects the levels of IL-6 in the supernatants of 10 NAs and CAAs with or without UCHL1 overexpression. (C) The interaction between UCHL1 and KLF5 was predicted by ZDOCK server, and the predicted complex structure was visualized using Discovery Studio software. (D) The interaction between UCHL1-KLF5 was confirmed by endogenous Co-IP. (E) Western blot shows the protein levels of UCHL1 and KLF5 after UCHL1 knockdown in CAA cells. (F) Following transfection with UCHL1 overexpression plasmids and treatment with MG132 for 4 h, CAA cell lysates were subjected to western blot. (G) Co-IP and western blot assays were used to evaluate the effect of UCHL1 overexpression on the ubiquitination of KLF5 proteins in HEK293T cells. (H) Co-IP and western blot assays were performed for lysates from HEK293T cells. (I-J) The expression of KLF5, IL-6, p65 NF-κB, p-p65 NF-κB, FABP4, adiponectin and perilipin in CAA cells with UCHL1 knockdown or KLF5 overexpression. (K) Images of subcutaneous mammary fat pad tumors in M-NSG mice in different groups. Tumors were harvested after 1.0×10^7^ MCF7 cells per mouse without or with 2.5×10^6^ CAAs (UCHL1 overexpression or knockdown) were injected into the subcutaneous mammary fat pad of the M-NSG mice. (L-M) Tumor weights of the M-NSG mice were recorded at the time of sacrifice, and a continuous line graph were applied. (N) The expression of p-p65 NF-κB, IL-6, perilipin and adiponectin in CAAs of different groups. The experiments were repeated twice with three biological replicates per experiment. *** *P*<0.001. independent two-sample Student's t test (Fig. 6B); One-way ANOVA followed by Tukey's test (Fig. 6L).

To identify potential deubiquitinases (DUBs) regulating IL-6 expression in CAA, RNA sequencing analysis was performed on CAA and NA samples. Next, by cross-referencing with the DUB gene set, UCHL1 mRNA level was found to be significantly upregulated in CAA (Figure 6A). ELISA test further in 10 NAs and CAAs confirmed that overexpression of UCHL1 increased the IL-6 level (Figure 6B). Co-IP coupled with LC-MS/MS was applied to screen UCHL1 interacting proteins, and KLF5, a critical upstream transcription factor of IL-6, was identified as the candidate target (Figure 6C). The three-dimensional UCHL1-KLF5 protein docking complex was predicted via Hdock, while co-IP assay validated their endogenous interaction (Figure 6D). Further experiments indicated that UCHL1 positively regulates KLF5 expression (Figure 6E, Supplementary Figure S14A), and MG132 treatment eliminated such upregulation, indicating proteasome-dependent regulation (Figure 6F). Further assays proved UCHL1 reduced overall and K48-linked ubiquitination of KLF5 (Figure 6G-H). UCHL1 silencing suppressed p65 phosphorylation and IL-6 level, yet raised adipocyte marker expression, which could be rescued by KLF5 overexpression (Figure 6I-J, Supplementary Figure S14B-C). *In vivo* assays demonstrated UCHL1 knockdown inhibited breast tumor growth, whereas UCHL1 overexpression exerted the opposite effect (Figure 6K-M). Detection of related biomarkers revealed UCHL1 activated NF-κB signaling and IL-6 production, and inhibited adipocyte differentiation (Figure 6N). Collectively, these results suggest that UCHL1 activates the p-p65/IL-6 pathway by stabilizing KLF5 through K48-linked deubiquitination, thereby promoting adipocyte dedifferentiation and exacerbating the development of breast cancer.

**Figure 7:**
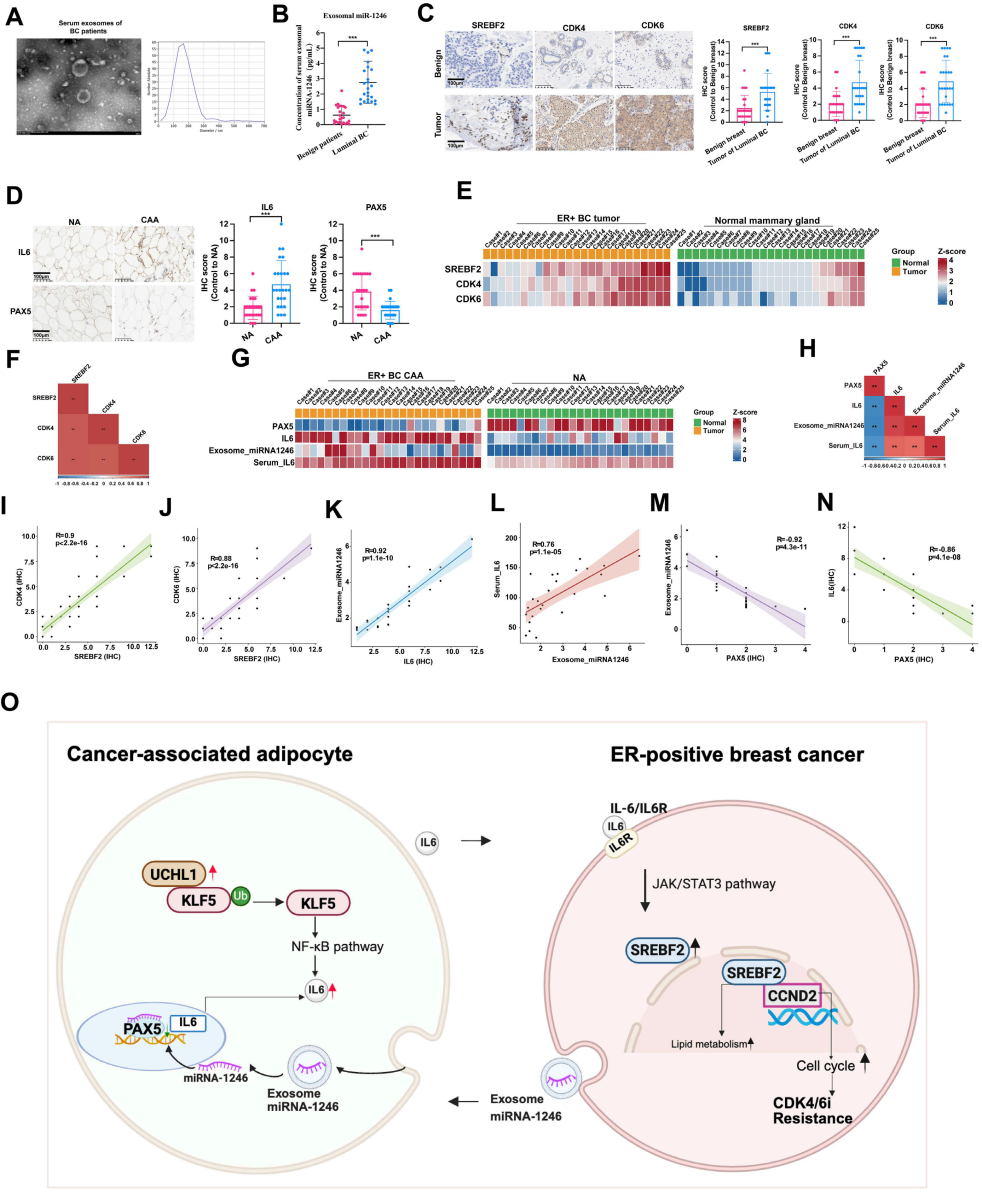
** Clinical sample validations.** (A) Exosomes derived from patients' serum were identified by electron microscope. (B) miR-1246 of serum were compared between the two groups. (C) The expression and statistical analysis of SREBF2, CDK4 and CDK6 in benign breast tissues and luminal breast cancer tumors. (D) The expression and statistical analysis of IL-6 and PAX5 in NAs and CAAs. (E) Heatmap of the expression of CDK4, CDK6 and SREBF2 across the 25 cases of benign breast tissues and 25 cases of luminal breast cancer tumors. (F) The correlations between CDK4 or CDK6 associated with SREBF2. (G) Heatmap of the expression of PAX5, IL-6 and miRNA-1246 across the 25 NAs and 25 CAAs samples. (H) The correlations between IL-6 or PAX5 associated with miRNA-1246. Positive correlation of CDK4 (I) or CDK6 (J) with SREBF2. Positive correlation of exosomal-miRNA-1246 with IL-6 (K), positive correlation of serum miRNA-1246 with IL-6 (L), and negative correlation of exosomal-miRNA-1246 (M) and serum miRNA-1246 (N) with PAX5. (O) This project systematically investigated the loop of CAAs-mediated resistance to CDK4/6i in ER-positive breast cancer. The experiments were repeated twice with three biological replicates per experiment. ** *P*<0.01, *** *P*<0.001. Mann-Whitney U (Fig. 7B, 7C, 7D).

Clinical correlation showed elevated serum exosomal miR-1246 in ER+ BC patients versus benign controls (Figure 7A-B). SREBF2, CDK4 and CDK6 expression were likewise higher in ER+ BC than normal tissue (Figure 7C). IL-6 expression was likewise higher while PAX5 expression was lower in CAAs versus NAs (Figure 7D). Correlation analysis validated positive correlations between SREBF2 and CDK4/CDK6 in ER-positive breast cancer (Figure 7E-F, 7I-J). By contrast, PAX5 expression in CAAs was negatively correlated with intratumoral IL-6, serum IL-6 and exosomal miR-1246 (Figure 7G-H, 7K-N). These findings demonstrate that ER+ BC-derived exosomal miR-1246 reprograms NAs into CAAs by suppressing PAX5, thereby upregulating IL-6 expression.

Collectively, this study reveals reciprocal crosstalk between CAAs and ER+ breast cancer cells that promotes CDK4/6i resistance. CAA-secreted IL-6 triggers JAK-STAT3 signaling and subsequently elevates SREBF2 transcription in tumor cells. SREBF2 exerts dual regulatory effects: it directly binds the CDKN2C promoter to facilitate cell cycle progression and also mediates cholesterol metabolic reprogramming. Multiple intervention assays including IL-6 neutralization, STAT3 suppression and SREBF2 knockdown validate that this axis is indispensable for the development of drug resistance.

The exosomal miR-1246-PAX5 and UCHL1-KLF5-NF-κB pathways do not mediate resistance separately. Instead, they converge to continuously boost IL-6 secretion in CAAs. Tumor-originated miR-1246 induces the transformation from NAs to CAAs via inhibiting PAX5 expression. UCHL1 stabilizes KLF5 through K48-linked deubiquitination, further enhancing NF-κB-mediated IL-6 transcription. These two upstream cascades jointly shape an inflammatory microenvironment and function as upstream regulators of the IL-6-STAT3-SREBF2 signaling cascade.

As illustrated in Figure 7O, the hierarchical regulatory network centers on the IL-6-STAT3-SREBF2-CDKN2C cascade as the core pathway. This core is reinforced by two upstream modules: the miR-1246-PAX5 axis and the UCHL1-KLF5-NF-κB axis. Together, this working model converges on SREBF2 and IL-6, identifying them as key nodes for clinical therapeutic intervention.

## Discussion

CDK4/6i combined with endocrine therapy constitutes the first-line treatment for advanced ER+ breast cancer 29. However, the inevitable emergence of acquired resistance underscores an urgent clinical need to decipher resistance mechanisms and develop novel therapeutic strategies 30. Growing evidence implicates multiple TME components, including cancer-associated fibroblasts, extracellular matrix (ECM) remodeling, adipocytes, and immune cells, in mediating such resistance 13, 31, 32. Among these, adipocytes represent a predominant TME population that critically influences tumor progression through cytokine secretion, angiogenic promotion, drug resistance facilitation, and immunosuppression 33, 34. Our study uncovers a bidirectional adipocyte-tumor metabolic axis that drives CDK4/6i resistance, characterized by: 1) CAAs as active contributors to CDK4/6i resistance; 2) CAAs promoting resistance via IL-6/JAK/STAT3 signaling, which activates SREBF2 to coordinate cholesterol metabolic reprogramming and cell cycle progression; and 3) tumor-secreted exosomal miR-1246 reprogramming normal adipocytes (NAs) into CAAs by suppressing PAX5, thereby creating an IL-6-enriched TME. Our data support CAA-derived IL-6 signaling through STAT3-SREBF2 as the core, non-cell-autonomous axis directly responsible for conferring CDK4/6i resistance; the exosomal miR-1246 and UCHL1/KLF5 pathways are demonstrated to be the upstream and sustaining mechanisms that establish and maintain the pathogenic CAA phenotype. These findings reveal a vulnerability that is therapeutically targetable to disrupt CDK4/6i resistance in ER+ breast cancer.

Adipocytes have evolved from being viewed as passive energy storage units to dynamic endocrine cells that secrete hormones, cytokines, and adipokines to regulate physiological and pathological processes 35. In the TME, malignant cells actively reprogram neighboring NAs through direct cellular crosstalk, driving their transdifferentiation into CAAs with distinct tumor-promoting properties. Through epigenetic and metabolic reprogramming, CAAs emerge as critical stromal components that support tumor progression. Our findings align with previous studies demonstrating that CAAs undergo multidimensional reprogramming in proximity to tumor cells, acquiring protumorigenic capabilities through coordinated cellular transformation 36. Breast cancer cells orchestrate NAs-to-CAAs conversion via secretory reprogramming, deploying paracrine effectors such as cytokines, growth factors, matrix-remodeling enzymes, and extracellular vesicles 37, 38. In turn, CAAs secrete elevated levels of cytokines, chemokines, and adipokines that promote tumor proliferation, dissemination, angiogenesis, and metastasis 39. Notably, CAAs also influence therapeutic response, contributing to treatment resistance 19, 40. While IL-6's role in inflammation and immune regulation is well-established 41, its specific involvement in CDK4/6i resistance requires further elucidation. Although not explicitly addressed in prior studies, emerging evidence as well as our work suggests that IL-6 may activate signaling pathways that modulate CDK4/6 activity, thereby promoting cell cycle progression and proliferation 42. Elevated IL-6 levels could potentially enable tumor cells to bypass CDK4/6-dependent cell cycle arrest through compensatory cyclin D-CDK6 activation or enhanced anti-apoptotic signaling 43. However, the precise mechanisms by which CAAs-derived IL-6 confers CDK4/6i resistance remain incompletely understood.

The development of CDK4/6i resistance represents a multifaceted biological process involving numerous molecular events. Emerging evidence highlights the critical role of cholesterol metabolism in tumor progression and therapeutic resistance 44. Our current study demonstrates that IL-6-induced activation of SREBF2 serves as a key regulator of both cholesterol metabolism and CDK4/6i resistance. As a master transcriptional regulator of sterol-responsive genes, SREBF2 plays pivotal roles in cholesterol biosynthesis, cellular metabolism, and oncogenesis 45, 46. While drug resistance mechanisms are context-dependent and vary across cancer types 47, accumulating evidence implicates SREBF2 in therapeutic resistance through diverse pathways. In hepatocellular carcinoma, caspase-3-mediated SREBF2 cleavage enhances cholesterol biosynthesis and cancer stemness, driving tyrosine kinase inhibitor resistance 21. In ovarian cancer, SREBF2 overexpression correlates with cisplatin resistance and cholesterol metabolic reprogramming 48. SREBF2-mediated transferrin regulation modulates intracellular iron pools, ROS levels, and lipid peroxidation, conferring resistance to ferroptosis inducers 49. Our findings reveal a novel mechanism whereby SREBF2 binds to CDKN2C to mediate CDK4/6i resistance. In addition, our findings identify a clinically actionable strategy: the HMGCR inhibitor simvastatin restores CDK4/6i sensitivity by targeting SREBF2-driven cholesterol biosynthesis downstream of IL-6-STAT3 signaling. Given their established safety and availability, statins are attractive for rapid repurposing. This approach may benefit patients with elevated IL-6 or SREBF2 expression, providing a mechanistic rationale for evaluating statins as adjunctive therapy in CDK4/6i-treated ER+ breast cancer.

Notably, our findings establish a mechanistically rational and clinically actionable rationale for integrating HMGCR inhibition by simvastatin and IL-6 blockade with CDK4/6i and standard endocrine therapy to reverse adipocyte-mediated therapy resistance. Adipose-rich TME, particularly those conditioned by NA or CAA, drive CDK4/6i resistance primarily through sustained secretion of IL-6, which activates downstream pro-survival and cell cycle bypass signaling that circumvents CDK4/6 inhibition. As a potent HMGCR inhibitor, simvastatin targets the SREBF2-HMGCR metabolic axis, not only suppressing mevalonate pathway activity but also disrupting the adipocyte inflammatory program that fuels IL-6 production and secretion. When combined with anti-IL-6 antibody, this dual intervention acts synergistically: statins abrogate the metabolic upstream trigger of adipocyte-derived inflammatory signaling, while IL-6 blockade directly neutralizes the key paracrine mediator of CDK4/6i resistance. This combinatorial strategy effectively restores sensitivity to CDK4/6i, enabling their cytostatic activity to cooperate with endocrine therapy in suppressing tumor cell proliferation. Given the well-established clinical safety, widespread availability, and low cost of statins, this dual targeting approach represents a highly translatable adjuvant strategy to overcome CDK4/6i resistance in adipocyte-rich malignancies, with immediate potential for clinical testing in combination with standard CDK4/6i and endocrine regimens. These findings underscore the importance of investigating the SREBF2 regulatory network in CDK4/6i resistance. Future studies should focus on elucidating the precise mechanisms of SREBF2-mediated resistance and developing targeted strategies against SREBF2 or its downstream effectors to overcome therapeutic resistance in cancer.

While research on direct reprogramming of NAs into CAAs remains limited, current evidence indicates this transformation requires coordinated genetic, transcriptional, and molecular alterations that distinguish CAAs from their normal counterparts through distinct gene expression profiles, enhanced secretory functions, and modified interactions with cancer cells 50. Our work identifies exosomal miR-1246 as a novel mediator of adipocyte reprogramming, building on previous reports of its roles in p53-mutant colorectal cancer 51, and correlates with tumor stage 52. In Helicobacter pylori-positive gastric cancer patients, tumor-derived exosomal miR-1246 can be transferred to lymphatic endothelial cells (LECs), promoting lymphangiogenesis and lymphatic remodeling 53. Functionally, miR-1246 downregulates key target genes involved in critical biological processes. Specifically, we identified PAX5 as a direct target of miR-1246, demonstrating its role in regulating both adipocyte differentiation and IL-6 secretion. As one of nine mammalian Pax transcription factors, PAX5 plays essential roles in early embryonic development and cellular differentiation 54, 55, particularly in driving immunophenotypic maturation and B-cell differentiation. Notably, PAX5 loss mediates plasma cell differentiation 54, highlighting its context-dependent functions. Furthermore, PAX5 participates in miRNA regulatory networks that influence tumor progression, exemplified by the PAX5-miR-142 negative feedback loop that modulates breast cancer progression through DNMT1 and ZEB1 targeting 56. Given the complexity of NAs-to-CAAs reprogramming, future studies should focus on elucidating the underlying molecular mechanisms and developing targeted therapeutic strategies against adipocyte-tumor crosstalk in the TME.

Our study reveals that the deubiquitinating enzyme UCHL1 is highly overexpressed in CAAs within the TME. We further elucidate a novel mechanistic pathway whereby UCHL1, by binding to and stabilizing the transcription factor KLF5, promotes the activation of the NF-κB signaling pathway, culminating in the upregulated expression and secretion of the key pro-inflammatory cytokine IL-6. This finding extends the functional repertoire of UCHL1 beyond its well-characterized roles in cancer cells themselves, positioning it as a critical regulator of the pro-tumorigenic phenotype of a major stromal component in breast cancer. The specific overexpression of UCHL1 in CAAs aligns with and refines emerging evidence on its role in the tumor stroma. Recent work has demonstrated that UCHL1 expression is elevated in adipose-derived stem cells and their exosomes under inflammatory stimulation, facilitating immune evasion in TNBC via the HDAC6/STAT3/PD-L1 axis 57. Our data pinpoint this dysregulation to the differentiated CAAs and delineate the cell-intrinsic mechanism driving their pro-inflammatory secretory profile, providing more direct cellular evidence for the central role of UCHL1 in stromal reprogramming. The core mechanistic contribution of this study is the establishment of the UCHL1-KLF5-IL-6 signaling axis in CAAs. We demonstrate that UCHL1 directly interacts with KLF5 and stabilizes it by countering its proteasomal degradation, a finding consistent with the report showing that UCHL1-mediated stabilization of KLF5 contributes to endocrine therapy resistance in TNBC cells 58. Our work significantly expands the biological consequence of this interaction from intrinsic tumor cell drug resistance to the extrinsic regulation of the inflammatory microenvironment. We provide the first evidence that in CAAs, KLF5, stabilized by UCHL1, acts as a co-activator for the NF-κB pathway. NF-κB is a master regulator of inflammation, and its activation directly transcribes IL-6 and other cytokines 59. While UCHL1 has been linked to NF-κB activation in macrophages 60, our study uniquely demonstrates this pathway in CAAs and identifies KLF5 as a crucial intermediary. This reveals a novel layer of regulation where UCHL1, via KLF5, potentiates NF-κB signaling to amplify the inflammatory output of CAAs. By defining the UCHL1/KLF5/NF-κB axis as a primary driver of IL-6 overexpression in CAAs, we position UCHL1 as a central inflammatory switch in these cells. Therefore, therapeutic targeting of the UCHL1-KLF5 axis represents a promising strategy to suppress this critical stromal support signal at its source.

Thus, our findings also suggest that tumors exhibiting high IL-6-JAK-STAT3 activity or SREBF2-dependent lipid metabolic reprogramming may be especially susceptible to combination approaches that address both cell-cycle control and niche-driven resistance. Accordingly, disrupting the upstream IL-6 paracrine loop with anti-IL-6 antibodies, and targeting the downstream metabolic effector SREBF2 with clinically available statins. This strategy directly engages the two key nodes of the resistance axis, potentially converting a pro-resistance adipocyte-rich microenvironment into a therapeutic vulnerability in ER-positive breast cancer. Patients with an activated IL-6/SREBF2 axis, which identified by elevated circulating IL-6/exosomal miR-1246 or tumor expression of SREBF2 target genes, are most likely to benefit from targeted interventions. Pharmacodynamic monitoring could include markers of HMGCR inhibition and reduced SREBF2 transcriptional activity; serum IL-6/soluble IL-6R levels may guide IL-6 blockade dosing, pending further validation. Upfront combination therapy with CDK4/6i, statins, and/or anti-IL-6 agents may be optimal, though adding simvastatin at progression is also supported by preclinical data and offers greater clinical feasibility with reduced upfront toxicities. We propose a phase Ib/II randomized trial in ER+ advanced breast cancer patients progressing on CDK4/6i plus endocrine therapy, comparing continued therapy alone vs. therapy plus simvastatin plus or minus anti-IL-6 antibody, with progression-free survival as the primary endpoint. A biomarker-enriched design is recommended to enhance statistical power and minimize sample size. These findings highlight the therapeutic potential of disrupting the interaction between ER+ breast cancer cells and CAAs as a promising strategy to overcome CDK4/6i treatment resistance.

Admittedly, the study also has some limitations. Firstly, we acknowledge that the number of PDO lines included in this study is relatively limited, which may constrain the generalizability of our findings. Therefore, conclusions drawn from the PDO experiments should be interpreted with appropriate caution. Nevertheless, the key observations from the organoid models were consistently recapitulated in the in vivo experiments and showed good alignment with the clinical data, supporting the robustness and translational relevance of our major conclusions. Secondly, although KEGG enrichment analysis implied the enrichment of cell adhesion-related pathways, these biological processes were not further verified by functional or protein-level experiments in the current study.

## Supplementary Material

Supplementary figures and tables, materials and methods.

## Data Availability

Data will be made available on request. Contact Prof. Xiaosong Chen, information: chenxiaosong0156@hotmail.com.
