# Supplementary material for: Cancer-associated adipocytes confer CDK4/6 inhibitor resistance in ER+ breast cancer through an IL-6/STAT3/SREBF2 axis coupled with cholesterol metabolism and cell cycle reprogramming
Source: Int J Biol Sci. 2026 Jun 10;22(11):6218–36. doi: 10.7150/ijbs.130653 (PMC13282890; doi:10.7150/ijbs.130653)
Supplement: Supplementary file 1 — Supplementary figures and tables, materials and methods. [file ijbsv22p6218s1.pdf]

Supplementary figures

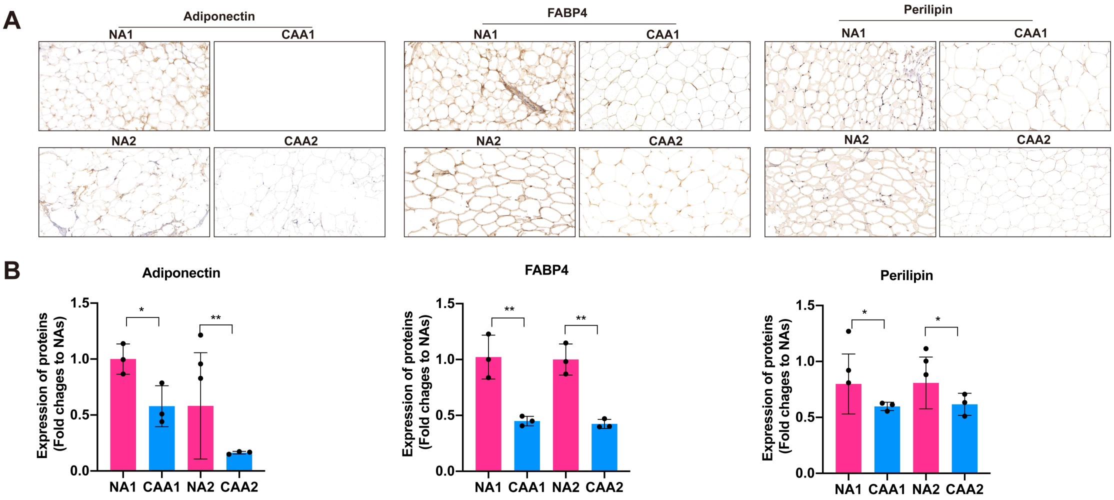

Figure S1. Decreased differentiation of CAAs. (A-B) IHC staining was performed to detect FABP4, Perilipin, Adiponectin expressions on NA and CAA tissues (200X). All results are expressed as mean ± SD. * *P*<0.05, ** *P*<0.01. Mann-Whitney U test (Fig. S1B).

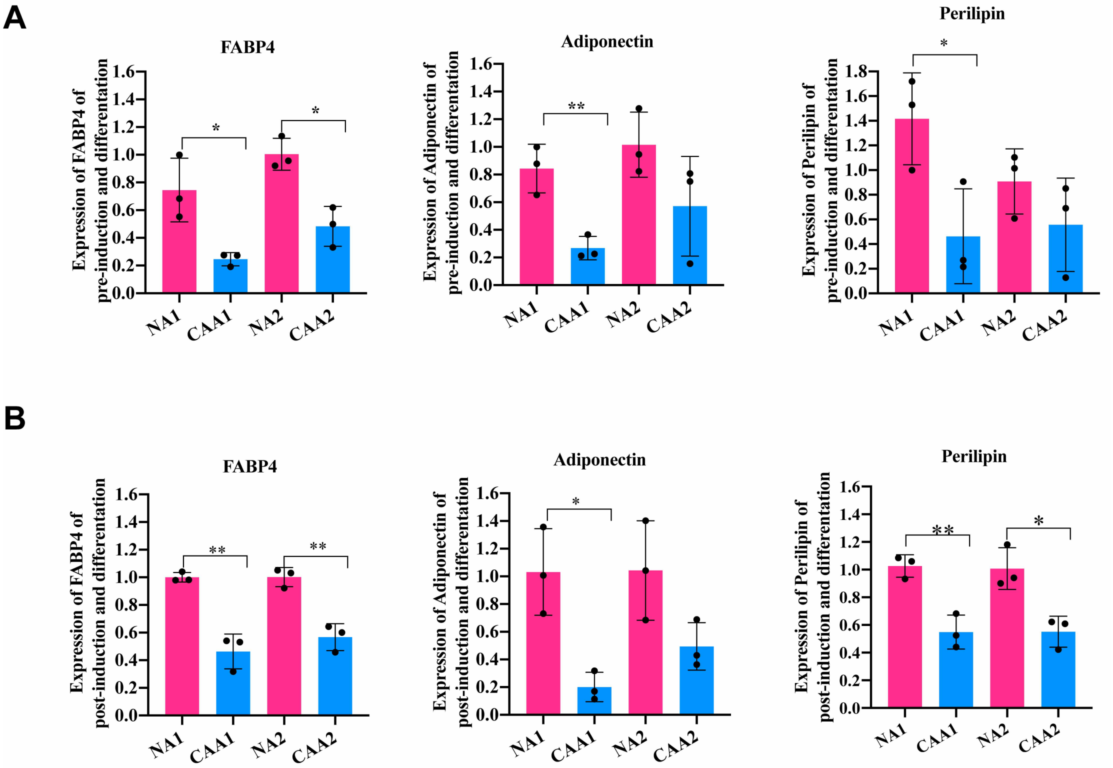

Figure S2. Decreased expression of adipose differentiation marker in CAA. (A) RT-PCR was used to identify FABP4, perilipin, adiponectin in adipocytes before induced differentiation of NAs and CAAs. (B) RT-PCR was used to identify FABP4, perilipin, adiponectin in adipocytes after induced differentiation for 28 days of NAs and CAAs. The experiments were repeated twice with three biological replicates per experiment. All results are expressed as mean ± SD. * P<0.05, ** P<0.01, *** P<0.001. independent two-sample Student's t test (Fig. S2A, S2B).

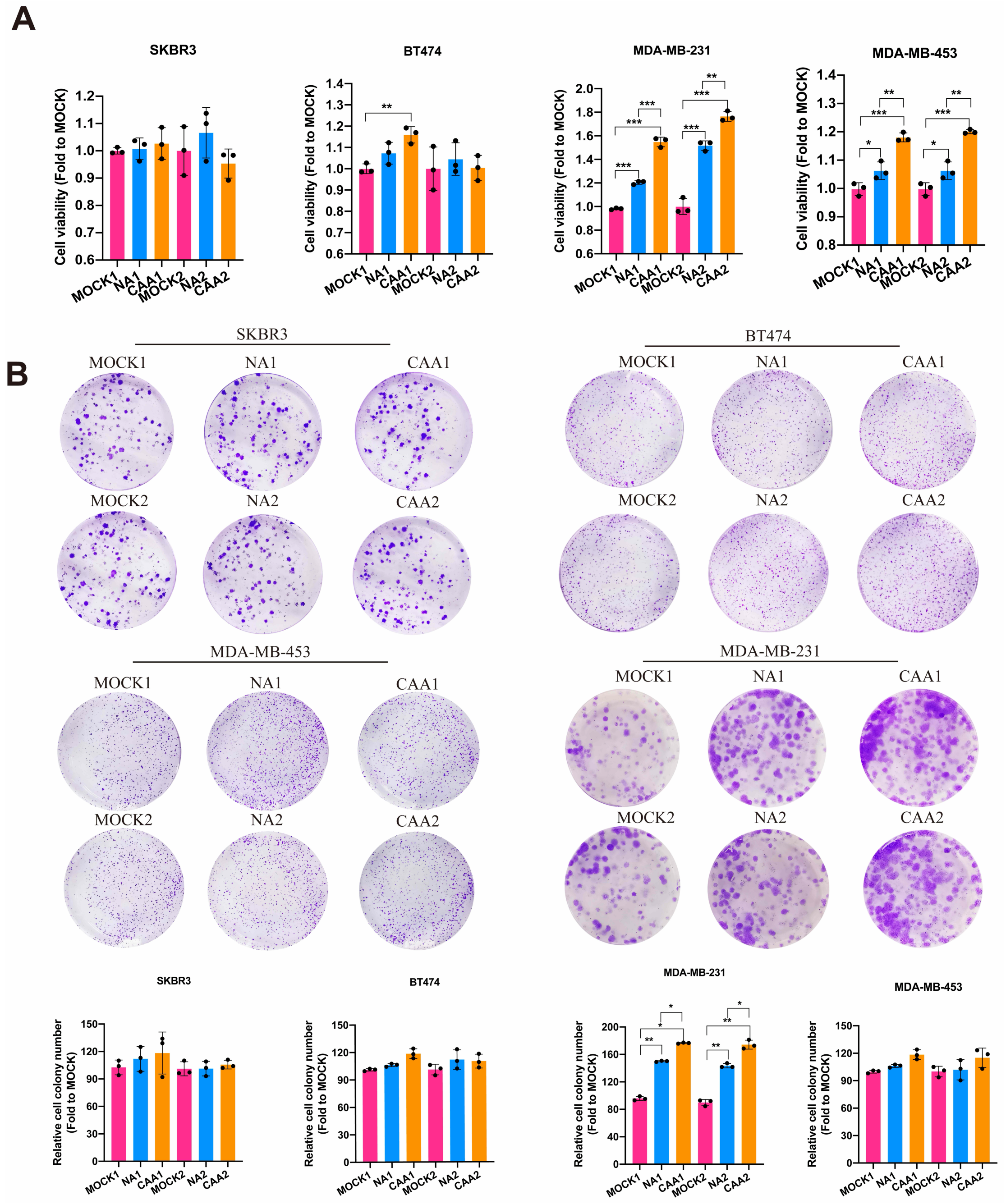

Figure S3. Role of NAs and CAAs in cell viability and colony formation in ER-positive, HER2 and TNBC. (A) The cell viability and statistical analysis of SKBR3, BT474, MDA-MB-453 and MDA-MB-231 cells after treated with NAs or CAAs supernatant for 7 days. (B) Colony formation assay shows the proliferative capacity of SKBR3, BT474, MDA-MB-453 and MDA-MB-231 cells after treated with NAs or CAAs supernatant for 14 days. The experiments were repeated twice with three biological replicates per experiment. All results are expressed as mean ± SD. * $P<0.05$, ** $P<0.01$, *** $P<0.001$. One-way ANOVA followed by Tukey's test (Fig. S3A, S3B).

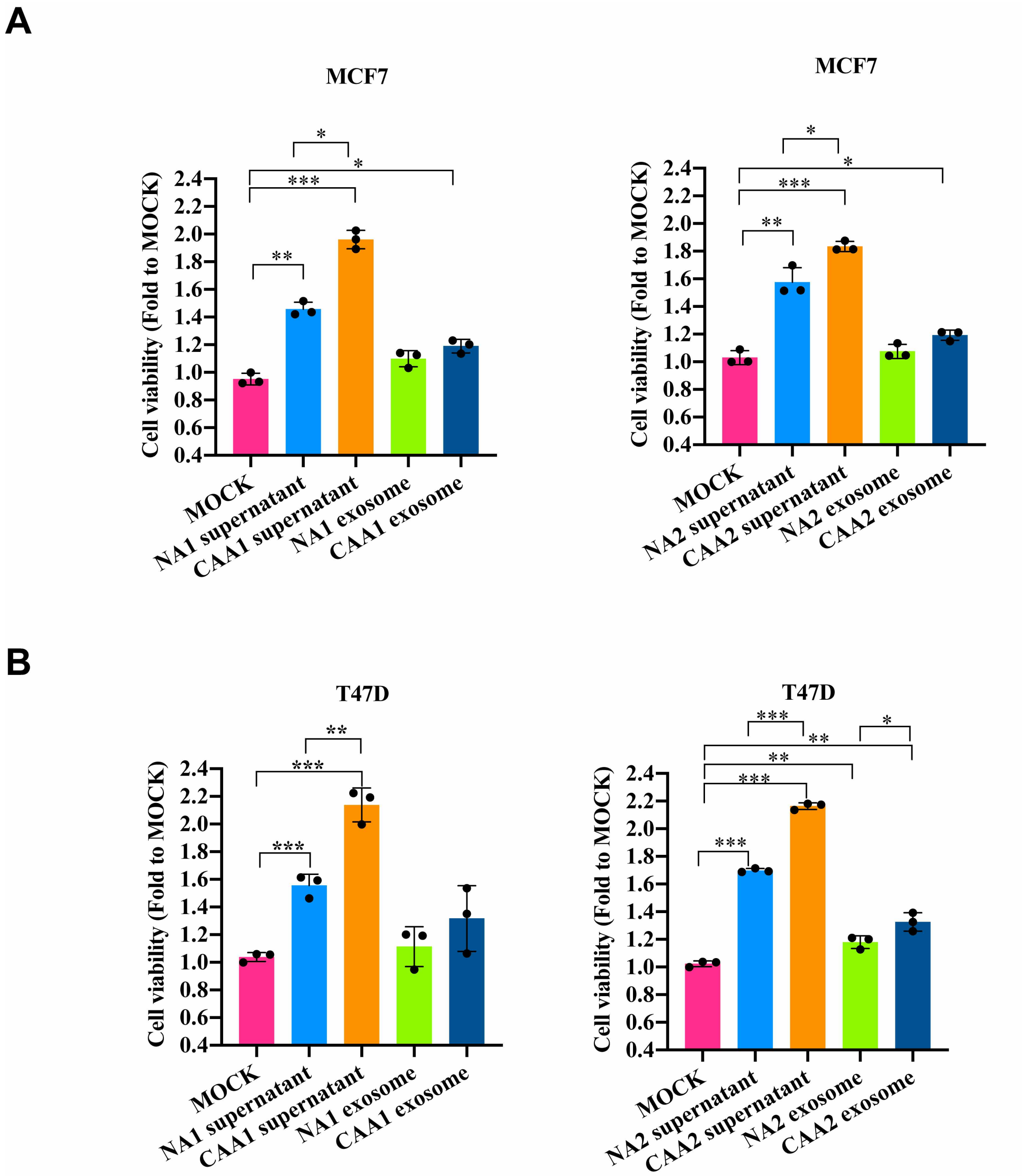

Figure S4. Role of the cellular supernatants of NAs and CAAs in cell viability. (A-B) The cell viability and statistical analysis of MCF7 and T47D cells after treated with NAs or CAAs supernatant (exosome-excluded) or exosomes from their supernatant for 7 days. The experiments were repeated twice with three biological replicates per experiment. All results are expressed as mean ± SD. * $P<0.05$, ** $P<0.01$, *** $P<0.001$. One-way ANOVA followed by Tukey's test (Fig. S4A, S4B).

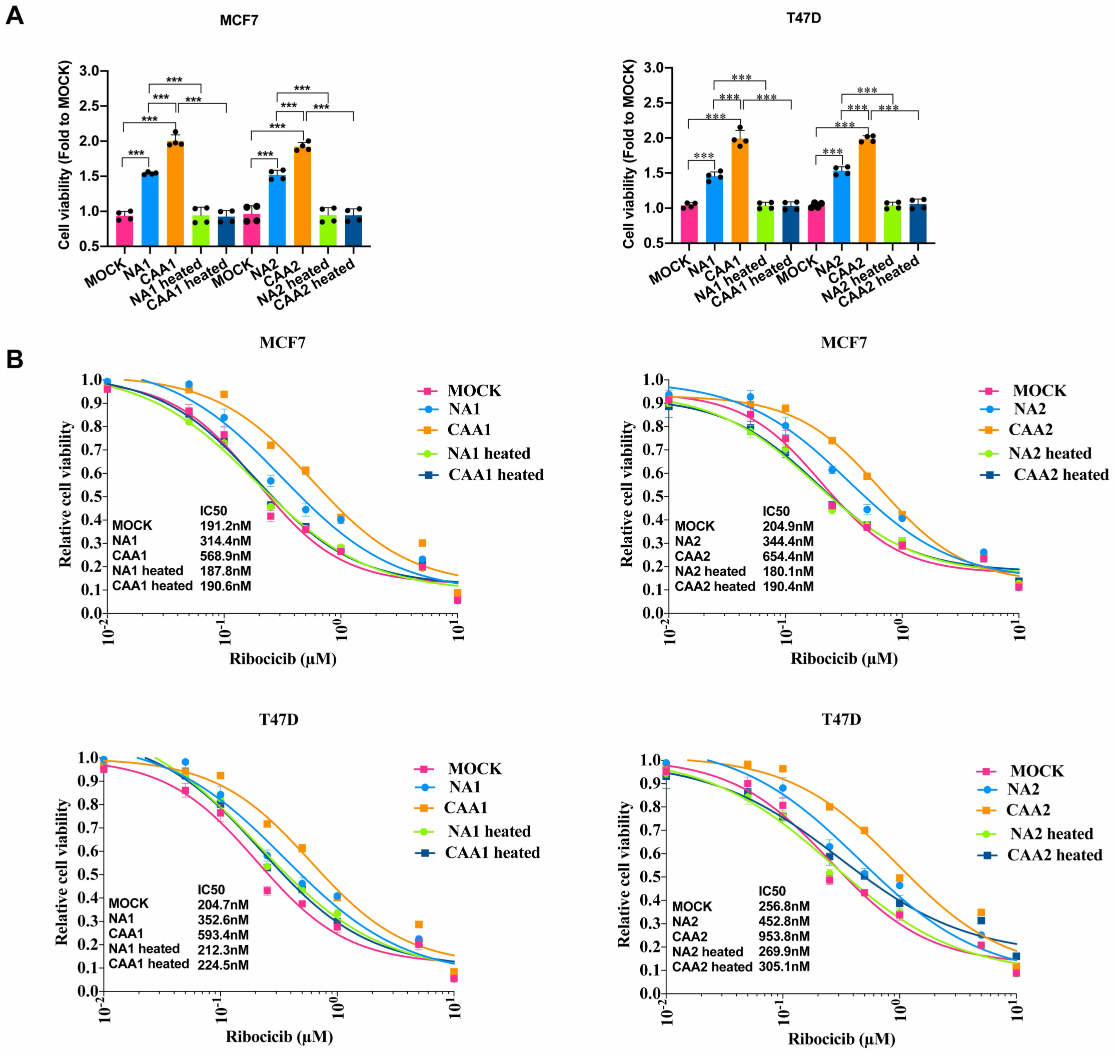

Figure S5. Cytokines in CAAs supernatant mediate cell proliferation and confers CDK4/6i resistance. (A) NAs or CAAs supernatant was heated at 95°C for 5 minutes to make cytokines or proteins inactive. Then, the cell viability of MCF7 and T47D cells were detected after treated with NAs or CAAs supernatant with or without heated for 7 days. (B) NAs or CAAs supernatant was heated at 95°C for 5 minutes to make cytokines or proteins inactive. CellTiter-Glo cell viability assay showed the survival rate and statistical analysis of IC50 of MCF7 and T47D cells induced with various concentrations of Ribociclib after treated with NAs or CAAs supernatant with or without heated for 7 days. The experiments were repeated twice with three biological replicates per experiment. All results are expressed as mean ± SD. *** P<0.001. One-way ANOVA followed by Tukey's test (Fig. S5A).

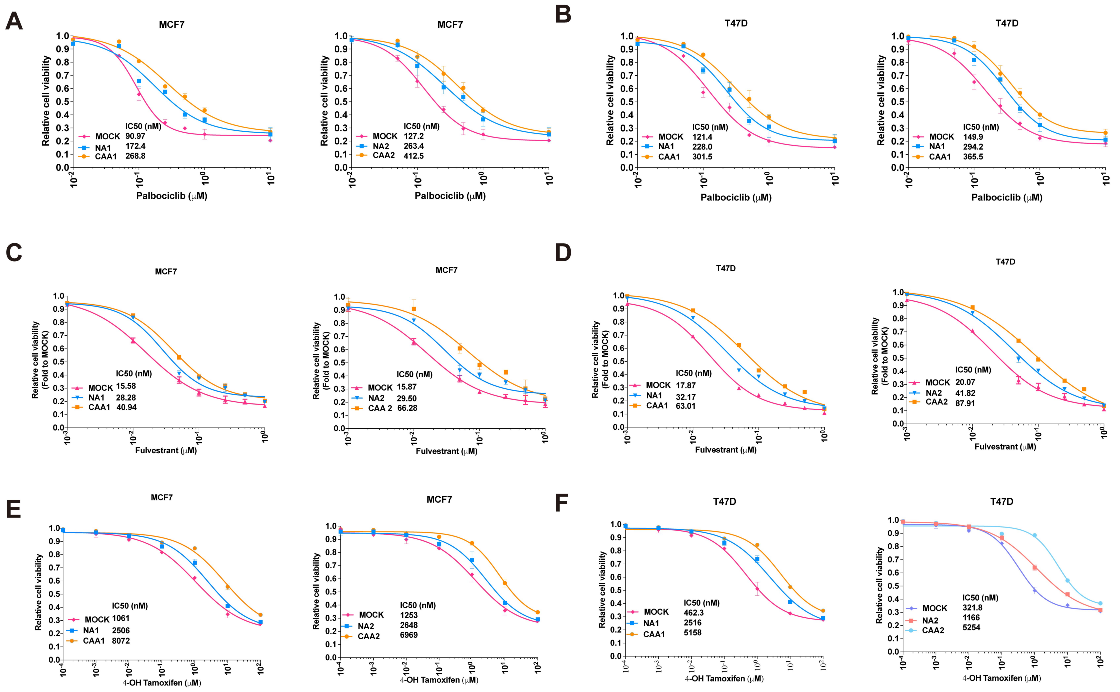

Figure S6. CAA confer Palbociclib, 4-OH Fulvestrant and Tamoxifen resistance. CellTiter-Glo cell viability assay showed the survival rate and statistical analysis of IC50 of MCF7 and T47D cells induced with various concentrations of Palbociclib (A-B), Fulvestrant (C-D) and 4-OH Tamoxifen (E-F) after treated with NAs or CAAs supernatant for 7 days. The experiments were repeated twice with three biological replicates per experiment.

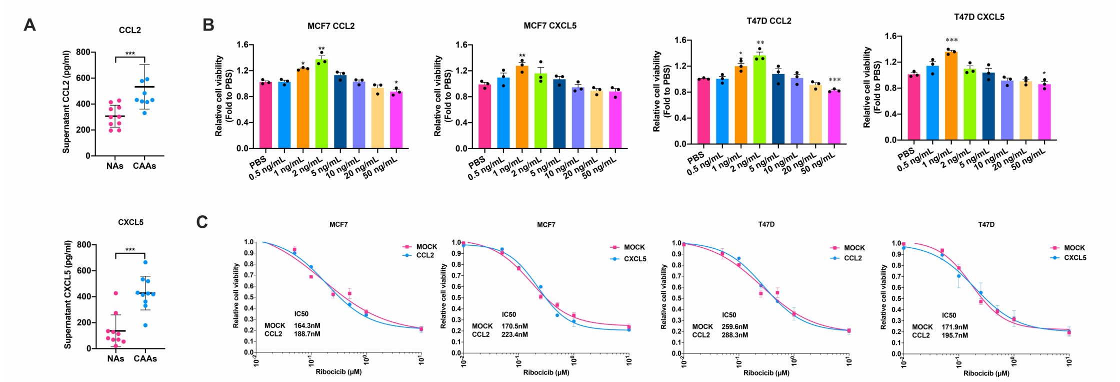

Figure S7. Effect of CCL2 and CXCL5 on tumor proliferation and Ribociclib resistance. (A) ELISAs detected CCL2 and CXCL5 secretory proteins in 10 NAs and 10 CAAs supernatants. (B) Cell viability of MCF7 and T47D cells after treated with different concentrations of CCL2 or CXCL5 for 7 days. (C) Cell viability assay depicted the survival rate and statistical analysis of IC50 of MCF7 and T47D cells induced with various concentrations of Ribociclib after treated with CCL2 (2 ng/ml) or CXCL5 (1ng/ml) for 7 days. * *P*<0.05, ** *P*<0.01, *** *P*<0.001. The experiments were repeated twice with three biological replicates per experiment. All results are expressed as mean ± SD. * P<0.05, ** P<0.01, *** P<0.001. independent two-sample Student's t test (Fig. S7A); One-way ANOVA followed by Tukey’s test (Fig. S7B).

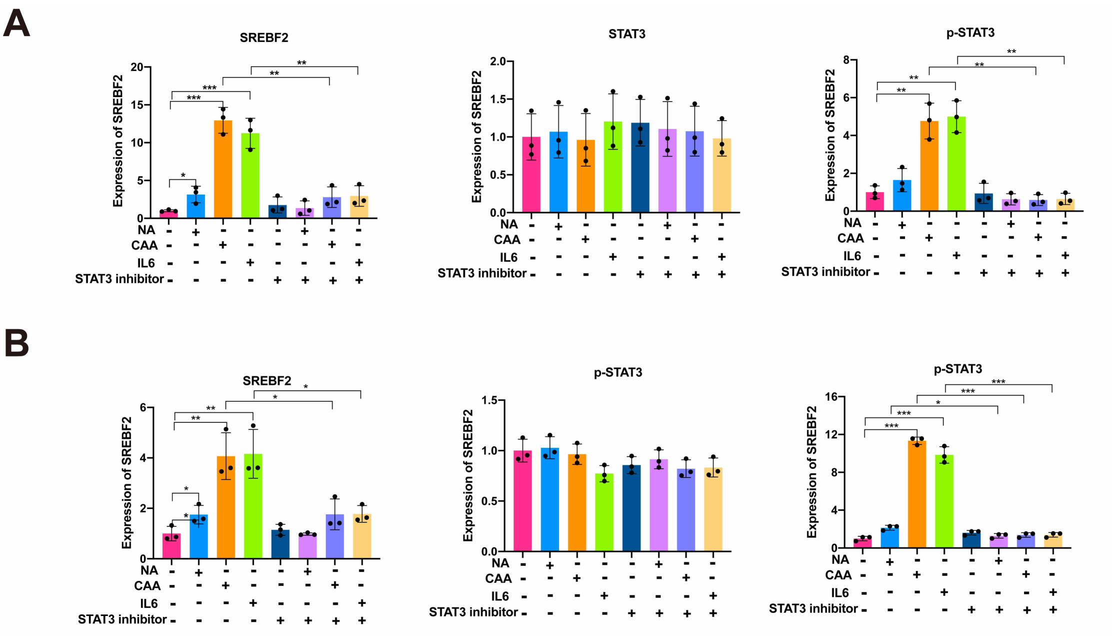

Figure S8. Statistical analysis of the proteins STAT3, p-STAT3 and SREBF2 of MCF7 (A) and T47D (B) cells with or without the treatment of NAs supernatants, CAAs supernatants, IL6 and STAT3 inhibitor WP1066. * *P*<0.05, ** *P*<0.01, *** *P*<0.001. The experiments were repeated twice with three biological replicates per experiment. All results are expressed as mean ± SD. * P<0.05, ** P<0.01, *** P<0.001. One-way ANOVA followed by Tukey's test (Fig. S8A, S8B).

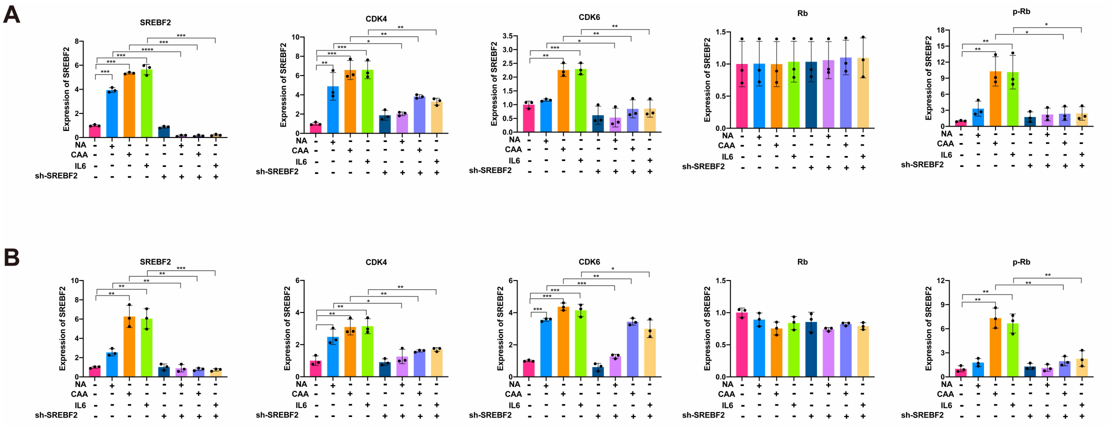

Figure S9. Statistical analysis of SREBF2, CDK4, CDK6, Rb and p-Rb in MCF7 (A) and T47D (B) cells with or without the treatment of NAs supernatants, CAAs supernatants, IL6 and SREBF2-knockdown. * *P*<0.05, ** *P*<0.01, *** *P*<0.001. The experiments were repeated twice with three biological replicates per experiment. All results are expressed as mean ± SD. * P<0.05, ** P<0.01, *** P<0.001. One-way ANOVA followed by Tukey's test (Fig. S9A, S9B).

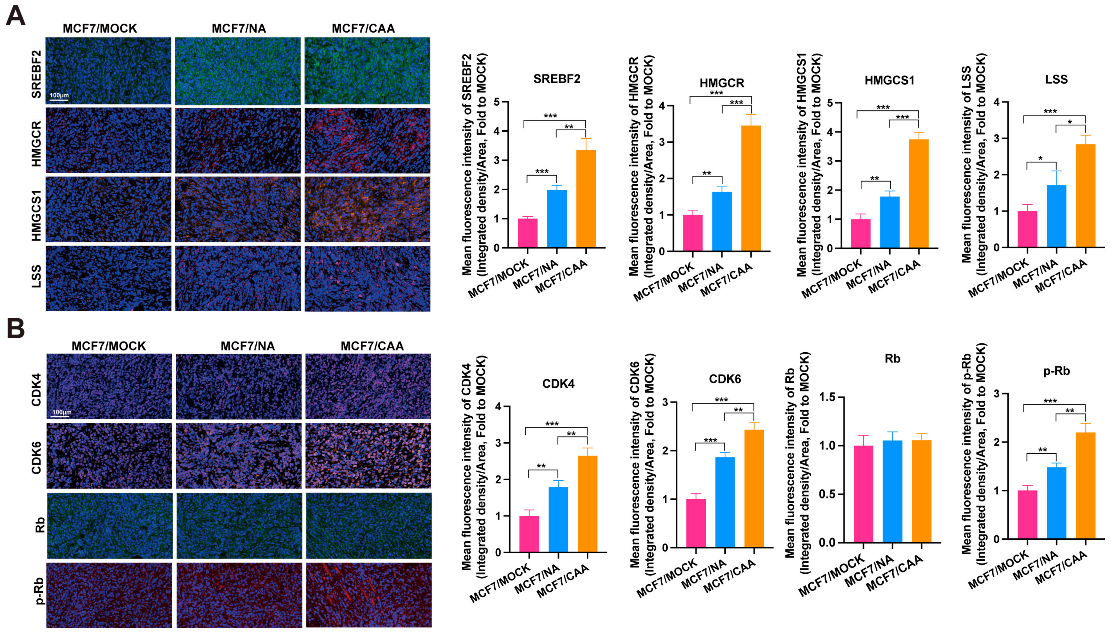

Figure S10. CAAs promote cell cycle and lipid metabolism *in vivo*. (A) IF staining and statical analysis of proteins SREBF2, HMGCR, HMGCS1 and LSS in the MCF7/MOCK, MCF7/NA, and MCF7/CAA groups. (B) IF staining and statistical analysis of proteins CDK4, CDK6, Rb and p-Rb in the MCF7/MOCK, MCF7/NA, and MCF7/CAA groups. The experiments were repeated twice with three biological replicates per experiment. All results are expressed as mean ± SD. * P<0.05, ** P<0.01, *** P<0.001. One-way ANOVA followed by Tukey's test (Fig. S10A, S10B).

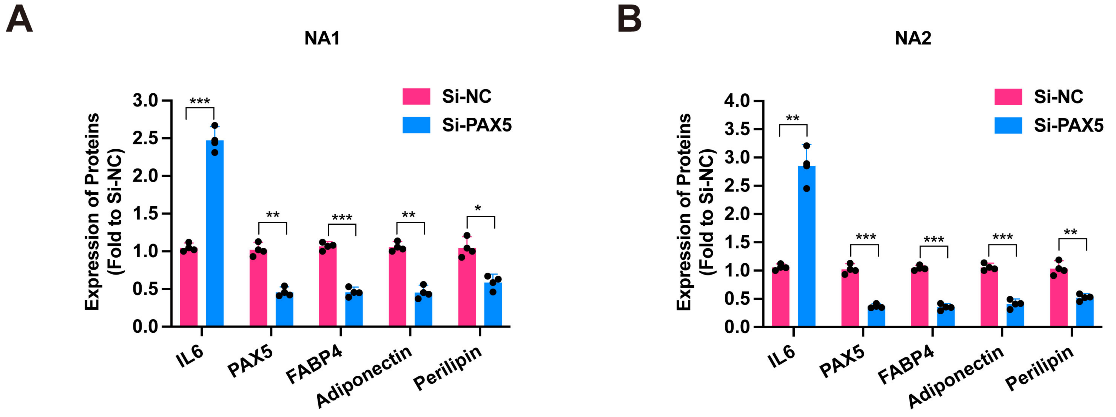

Figure S11. PAX5 mediates NAs reprogrammed into CAAs. Western blot statistical analysis of the expression of FABP4, perilipin, adiponectin in NA1 and NA2 with or without PAX5 knockdown. The experiments were repeated twice with three biological replicates per experiment. All results are expressed as mean ± SD. * P<0.05, ** P<0.01, *** P<0.001. independent two-sample Student's t test (Fig. S11A, S11B).

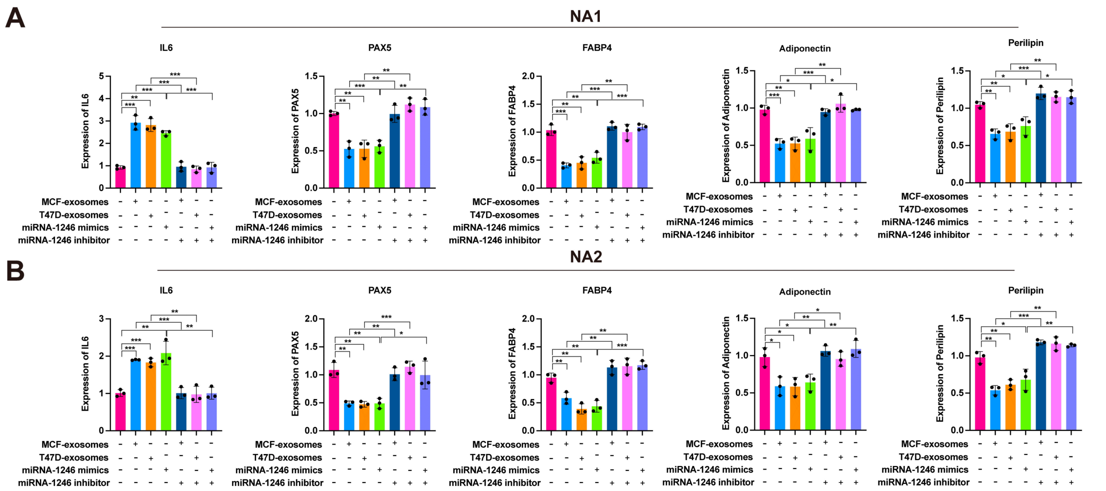

Figure S12. Tumor cell-derived exosomal miRNA-1246 decreased the differentiation of CAAs. (A-B) Western blot statistical analysis of the expression of FABP4, perilipin, adiponectin in NAs cells with the treatment of MCF7 exosomes, T47D exosomes, miR-1246 mimics, with or without miR-1246 inhibitor in NA1 and NA2 cells. The experiments were repeated twice with three biological replicates per experiment. All results are expressed as mean ± SD. * P<0.05, ** P<0.01, *** P<0.001. One-way ANOVA followed by Tukey's test (Fig. S12A, S12B).

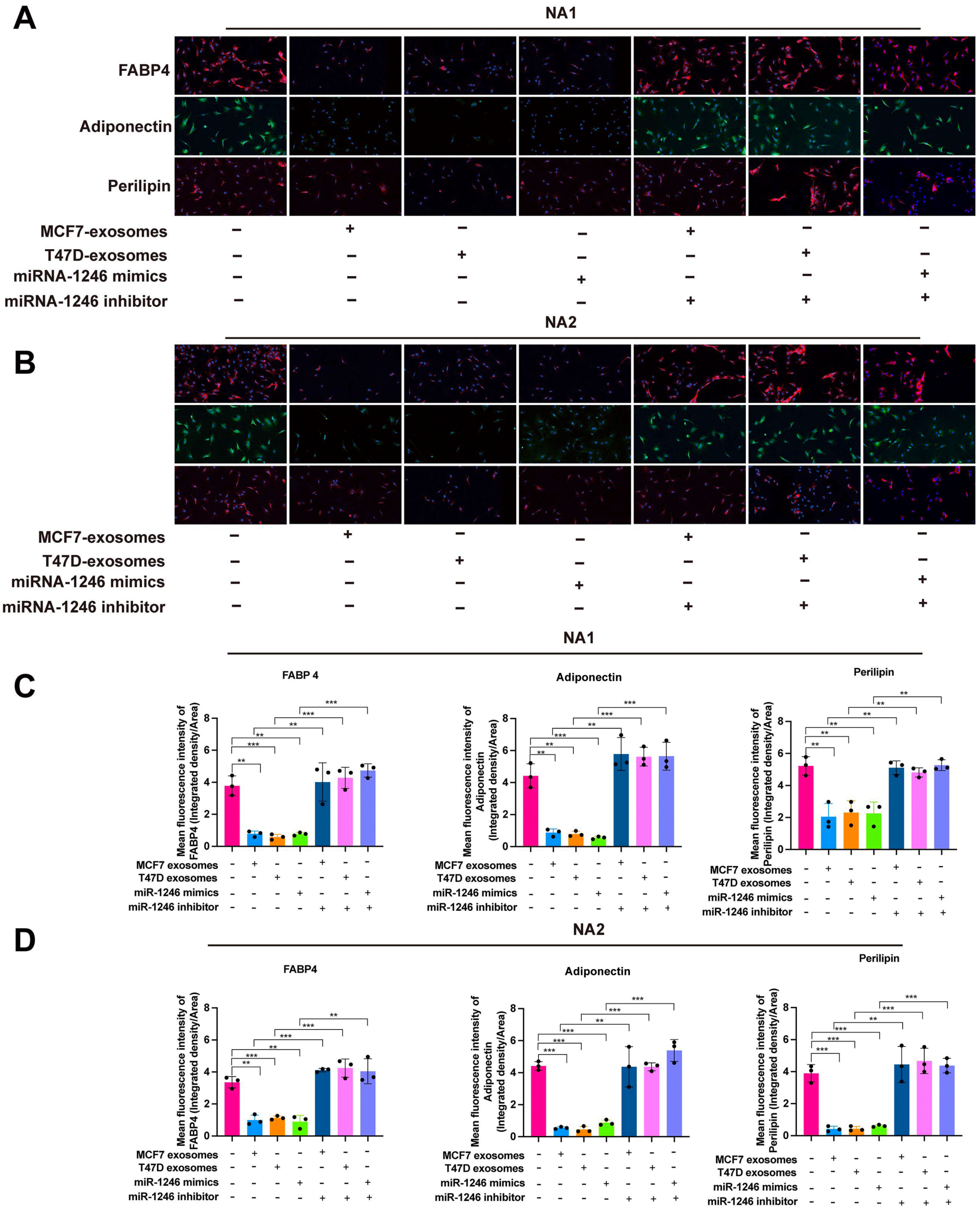

Figure S13. Tumor cell-derived exosomal miRNA-1246 decreased the differentiation of CAAs. IF (A-B) and its statistical analysis (C-D) of the expression of FABP4, perilipin, adiponectin in NAs with the treatment of MCF7 exosomes, T47D exosomes, miR-1246 mimics, with or without miR-1246 inhibitor in NA1 and NA2 cells. The experiments were repeated twice with three biological replicates per experiment. All results are expressed as mean ± SD. ** P<0.01, *** P<0.001. One-way ANOVA followed by Tukey's test (Fig. S13C, S13D).

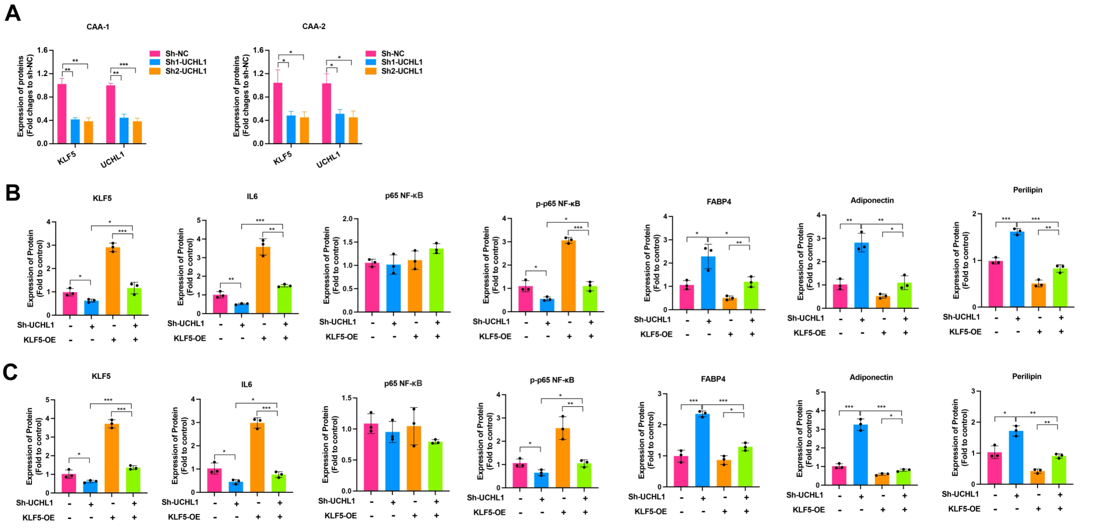

Figure S14. (A) Western blot statistical analysis of UCHL1 and KLF5 proteins after UCHL1 knockdown in CAA cells. (B) Western blot statistical analysis of KLF5, IL6, p65 NF-κB, p-p65 NF-κB, FABP4, adiponectin and perilipin proteins in CAA cells with UCHL1 knockdown or KLF5 overexpression. The experiments were repeated twice with three biological replicates per experiment. All results are expressed as mean ± SD. * P<0.05, ** P<0.01, *** P<0.001. One-way ANOVA followed by Tukey's test (Fig. S14A, S14B, S14C).

Supplementary tables

Table S1. Human obesity antibody array for the semi-quantitative detection of 62 human proteins.

	A	B	C	D	E	F	G	H	I	J	K	L	M	N
**1**<br>**2**	POS	POS	NEG	NEG	4-1BB	ACE-2	Adiponectin (ACRP30)	Adipsin	AgRP	ANGPT2	ANGPTL1	ANGPTL4	CRP	ENA-78 (CXCL5)
**3**<br>**4**	FAS (Apo-1)	FGF-6	Growth hormone	HCG-4 (CCL16)	IFN-γ	IGFBP-1	IGFBP-2	IGFBP-3	IGF-1	IGF-1R	IL-1R4 (ST2)	IL-1RI	IL-10	IL-11
**5**<br>**6**	IL-12 p70	IL α (IL-1F1)	IL-1β (IL-1F2)	IL-6	IL-6R	IL-8 (CXCL8)	Insulin	IP-10 (CXCL10)	Leptin R	Leptin	LIF	Lymphotactin	MCP-1 (CCL2)	MCP-3 (CCL7)
**7**<br>**8**	M-CSF	MIF	MIP-1 beta (CCL4)	MSP α/β	OPG	OSM	PAI-1	PARC (CCL18)	PDGF-AA	PDGF-AB	PDGF-BB	RANTES (CCL5)	Resistin	SAA
**9**<br>**10**	SDF-α	TNF RII	TNF RI	TECK (CCL25)	TGF β1	TIMP-1	TIMP-2	TNF alpha	VEGF-A	XEDAR	BLANK	BLANK	BLANK	POS

Table S2. Primary antibodies for western blot, immunofluorescence, immunohistochemistry.

Protein	Concentration Western blot	Concentration IF	Concentration IHC	Specificity	Company Lot No.
Adiponectin	/	1:1000	/	Rat anti-human	Abcam Cat#313402
Adiponectin	/	/	1:50	Rat anti-human	Novus Biologicals Cat# NBP2-22450
FABP4	/	1:1000	1:50	Rat anti-human	Abcam Cat# ab92501
Perilipin	/	1:1000	/	Rat anti-human	Abcam Cat# ab172907
Perilipin	/	/	1:50	Rat anti-human	Abcam Cat# ab3526
SREBF2	1:1000	1 µg/ml	1:50	Rat anti-mouse or human	Abcam Cat# ab30682
CDK4	1:1000	1:1000	1:200	Rat anti-human	Cell Signaling Technology Cat# 23972
CDK6	1:1000	1:1000	1:200	Rat anti-human	Cell Signaling Technology Cat# 23972
RB	1:1000	1:1000	1:200	Rat anti-human	Cell Signaling Technology Cat# 23972
P-RB	1:1000	1:1000	1:200	Rat anti-human	Cell Signaling Technology Cat# 23972
STAT3	1:1000	1:1000	1:200	Rat anti-human	Cell Signaling Technology Cat# 23972
P-STAT3	1:1000	1:1000	1:200	Rat anti-human	Cell Signaling Technology Cat# 23972
IL6	1:1000	1:1000	1:200	Rat anti-human	Abcam Cat# 23972
GAPDH	1:1000	/	/	Rat anti-mouse or human	CST Cat# 5174

IF: immunofluorescence; IHC: immunohistochemistry.

Table S3. Primers of genes for RT-qPCR applied in this study.

Gene	Forward sequence	Reverse sequence

Perilipin	TGTGCAATGCCTATGAGAAGG	AGGGCGGGGATCTTTTCCT
Adiponectin	TGCTGGGAGCTGTTCTACTG	TACTCCGGTTTCACCGATGTC
FABP4	ACTGGGCCAGGAATTTGACG	CTCGTGGAAGTGACGCCTT
PAX5	GTCACAGCATAGTGTCCACTG	CCGCTGATGGAGTACGACG
SREBF2	GACAGACGCCAAGATGCACAA	TCAGAGTCAATGGAGTAGGGAG
ELOVL5	TAACAGGAGTATGGGAAGGCA	ACCAGAGGACACGGATAATCTT
HLA-DRA	AGTCCCTGTGCTAGGATTTTTCA	ACATAAACTCGCCTGATTGGTC
POR	GGTGGCCGAAGAAGTATCTCT	AACCAGTAGGTTAGGAGACCC
LSS	GGCAGACGTGGACCTACCT	GAAAAGTGGGCCACCATAATC
TMEM132A	CTAGACGCCCCTGAACACTTC	GTGGCAAAAGGTGGGTAGGA
DHCR24	GCCGCTCTCGCTTATCTTCG	GTCTTGCTACCCTGCTCCTT
FASN	AAGGACCTGTCTAGGTTTGATGC	TGGCTTCATAGGTGACTTCCA
NBEAL2	GCTACTTCCATGTCCTTAATGCT	TGAGCCGAGTGAGGTGTTCA
GAPDH	GGAGCGAGATCCCTCCAAAAT	GGCTGTTGTCATACTTCTCATGG

CXCL8: C-X-C motif chemokine ligand; RT-qPCR: Real-time quantitative polymerase chain reaction.

Table S4. Primers of miRNAs applied in this study.

miRNAs	Sequence
hsa-miR-21-5p RT	GTCGTATCCAGTGCAGGGTCCGAGGTATTCGCACTGGATACGACTCAACA
hsa-miR-21-5p F	CCGCCGTAGCTTATCAGACTG
hsa-miR-1246 RT	GTCGTATCCAGTGCAGGGTCCGAGGTATTCGCACTGGATACGACAACAGG
hsa-miR-1246 F	CCGCCGCAAGTCTTATTTGAGC
hsa-miR-122-5p RT	GTCGTATCCAGTGCAGGGTCCGAGGTATTCGCACTGGATACGACCAAACA
hsa-miR-122-5p F	GCCGTGGAGTGTGACAATGG
hsa-miR-451a RT	GTCGTATCCAGTGCAGGGTCCGAGGTATTCGCACTGGATACGACAACTCA
hsa-miR-451a F	CGCCGAAACCGTTACCATTAC
hsa-miR-3622a-5p RT	GTCGTATCCAGTGCAGGGTCCGAGGTATTCGCACTGGATACGACCTCACC
hsa-miR-3622a-5p F	GCAGGCACGGGAGCTCA
hsa-miR-142-3p RT	GTCGTATCCAGTGCAGGGTCCGAGGTATTCGCACTGGATACGACTCCATA
hsa-miR-142-3p F	CGCCGTGTAGTGTTTCCTACTT
hsa-miR-1290 RT	GTCGTATCCAGTGCAGGGTCCGAGGTATTCGCACTGGATACGACTCCCTG
hsa-miR-1290 F	CCGCCGTGGATTTTTGGATC
hsa-miR-144-3p RT	GTCGTATCCAGTGCAGGGTCCGAGGTATTCGCACTGGATACGACAGTACA
hsa-miR-144-3p F	CGCCGCCGTACAGTATAGATGA
hsa-miR-126-3p RT	GTCGTATCCAGTGCAGGGTCCGAGGTATTCGCACTGGATACGACCGCATT
hsa-miR-126-3p F	CGCCGTCGTACCGTGAGTAAT
UnivR	AGTGCAGGGTCCGAGGTATT

Supplementary methods

Isolation and induction of adipocytes

NAs and CAAs were isolated from tissue samples within 2 cm of tumors in patients with benign breast conditions and breast cancer, respectively, using established protocols [24]. Briefly, adipose tissues were washed with Phosphate-Buffered Saline (PBS), minced into fragments, and digested with 0.1% type II collagenase (Sigma-Aldrich) at 37°C for 30 min with gentle agitation. The digestion was terminated by adding complete medium, and the cell suspension was filtered through a 40-μm nylon mesh (BD Biosciences) before centrifugation at 300 × g for 5 min to collect the stromal vascular fraction. Isolated cells were plated in DMEM/F12 medium supplemented with 10% fetal bovine serum (FBS), 1% penicillin/streptomycin, and 1 mM L-glutamine, and maintained at 37°C in 5% CO_2. To induce differentiation, primary preadipocytes were treated for 4 days with differentiation cocktail containing 5 μg/ml insulin, 1 μM dexamethasone, and 0.5 mM IBMX (both from Sigma-Aldrich), followed by 4 days in maintenance medium with 5 μg/ml insulin. Successful differentiation was confirmed by characteristic lipid-laden morphology and positive Oil Red O staining after 30-min incubation.

Establishment of mice model

Five- to six-week-old female wild-type nude mice were obtained from Shanghai SLAC Co., Ltd. (Shanghai, China), with all experimental procedures approved by the Institutional Animal Care and Use Committee of Shanghai Jiao Tong University School of Medicine (Animal ethics approval number：JUMC2023-184-A). To assess NAs and CAAs effects on Ribociclib resistance, mice received subcutaneous injections containing 1.0×10^7 MCF7 cells (with or without SREBF2 knockdown) combined with 2.5×10^6 NAs or CAAs. Following tumor establishment (50 mm^3), Ribociclib was administered every other day (Ribociclib 100 mg/kg). At week seven, tumors were surgically excised with normal tissue margins, while lung tissues and subcutaneous tumors were collected for HE stains, IF, or IHC analysis. To assess the combination

effect of anti-IL-6 and Simvastatin, the mice were injected with either the anti-IL-6 antibody (10 μg once) and/or Simvastatin (10 mg/kg once) by oral gavage, which scheduled every two days. Tumors were harvested after 1.0×10^7 MCF7 cells per mouse were injected into the subcutaneous mammary fat pad of the M-NSG mice. To evaluate the function of exosomal derived miR-1246 to CAA, mammary fat pad tumors in M-NSG mice in the MCF7 (mice received subcutaneous injections containing 1.0×10^7 MCF7 cells), MCF7/CAA (mice received subcutaneous injections containing 1.0×10^7 MCF7 cells with 2.5×10^6 CAAs), MCF7/CAA with the treatment of exosomes isolation from MCF7 cell culture supernatants, MCF7/CAA with the treatment with miRNA-1246 mimics or inhibitor. Tumor weights of the M-NSG mice were recorded at the time of sacrifice, and a continuous line graph were applied to compare tumor growth in each group of the M-NSG mice every 10 days.
